# Value driven co-design of smart sportswear for heat illness prevention in tropical regions

**DOI:** 10.3389/fdgth.2026.1872925

**Published:** 2026-08-18

**Authors:** Tharushi Wickramarathne, Blair Kuys, Wei Liu, Abdullah Al Mahmud

**Affiliations:** 1 Centre for Design Innovation, Swinburne University of Technology, Melbourne, VIC, Australia; 2 Faculty of Design, University of Macau, Avenida da Universidade, Taipa, Macao SAR, China

**Keywords:** co-design, endurance cycling, e-textiles, heat illness prevention, heat stress, human-centered design, low-resource settings, smart sportswear

## Abstract

**Background:**

Heat illness poses a serious health risk for endurance athletes in tropical climates, yet smart sportswear solutions integrating wearable digital health functions remain underexplored in low-resource settings.

**Objective:**

The objective of this research was to co-design concepts for wearable technology-enabled cycling sportswear that integrate cooling, physiological monitoring, culturally responsive usability, and cost-sensitive implementation to inform heat illness prevention strategies and support future prototype development.

**Methods:**

We conducted five co-design workshops with 20 semi-professional cyclists in Sri Lanka, using the Functional–Expressive–Aesthetic (FEA) framework to elicit user needs. Participants generated 19 design concepts and prioritized features through group discussion, sticky-note voting and qualitative ranking based on perceived health impact, usability, and cost feasibility.

**Results:**

Cooling comfort emerged as the top priority, with strong preference for hybrid designs combining natural wind cooling and smart user-controllable cooling. Key cooling zones included neck/upper back and underarm/chest. Participants valued physiological monitoring when it supported actionable interventions, such as future sensor-triggered cooling and hydration prompts, rather than passive monitoring dashboards. Additional priorities included localized UI in Sinhala, simplified data visualization, and safety features such as reflective elements. Two T-shirt concepts were finalized: smart cooling with user-controlled settings and lightweight cooling packs integrated with perforated panels.

**Conclusions:**

Findings suggest that smart sportswear for low-resource tropical cycling contexts should prioritise actionable, localised, and cost-sensitive cooling interventions rather than monitoring-only functions. The study contributes concept-level design requirements for culturally responsive wearable digital health sportswear. Because the findings are based on co-design outputs and participant perceptions, the proposed health benefits remain conceptual and require prototype development, physiological validation, usability testing, and field evaluation under cycling conditions.

## 1 Introduction

Heat illness is a serious public health issue among endurance athletes, especially those participating in sports in tropical environments where high temperatures and humidity pose an increased physiological burden (1, 2). The use of digital health technologies offers potential solutions through continuous monitoring and early identification, thus facilitating fast intervention using wearable systems (3). However, most of the smart clothing technologies available on the market have been designed for athletes in well-resourced regions, thus failing to take account of the unique problems experienced by athletes in developing nations such as lack of cooling apparatus, communication difficulties, and financial constraints (4). As global temperatures continue to rise and heatwaves intensify, heat strain is becoming a growing concern not only in low-resource tropical regions but also in developed countries, underscoring the need for effective, accessible cooling interventions (5). The inadequacy of available technology requires designers to embrace a human-centered approach that combines wearable health technologies with sportswear customized to specific cultures and climates. By integrating wearable physiological sensors, personal cooling technologies, and emergency communication features into cycling sportswear, smart sportswear can evolve from being mere performance enhancement gadgets to wearable healthcare technologies (6).

Researchers have explored smart wearable technologies to protect individuals from adverse health conditions, and these technologies can be used to minimize heat-related health concerns experienced by athletes (3, 7). However, most of these studies have focused on mid- and high-resource countries, overlooking disadvantageous athletes living in low-resource developing countries. Nevertheless, low-income and marginal groups have been identified as susceptible to heat illnesses (4, 8, 9) and the scarcity of cooling products/devices in developing communities, which can minimize the risk of heat illnesses, could be one reason for this situation (10). Consequently, individuals in developing and tropical countries such as Sri Lanka, India, and Thailand are at a higher risk of heat illness (5, 11, 12). It is important to explore how smart wearable technologies such as smart garments can minimize health concerns, particularly from the heat experienced by sportspersons, focusing on disadvantaged and overlooked athletes living in developing countries.

This research aims to fill this gap through an analysis of how human-centered design and co-design techniques can be applied to incorporate health wearable technology into clothing that is sensitive to cultural and environmental conditions. The three key considerations in this context include: (1) use of smart cooling systems to lower heat stress, (2) monitoring of physiology and emergency communication for health security, and (3) usability aspects. The purpose of this research was to co-design smart cycling sportswear that combines digital health functions with value-driven design principles to prevent heat illness among endurance athletes in tropical regions. By embedding sensors, personalized cooling mechanisms, and communication features, this work positions smart sportswear as a connected health intervention (13–15).

This study contributes to smart sportswear and wearable digital health research in three ways. First, it addresses the underexplored context of endurance cycling in a tropical, low-resource setting, where heat, humidity, affordability, and language accessibility shape technology acceptance. Second, it shifts the design emphasis from monitoring-only smart garments toward cooling-focused and action-oriented concepts that participants perceived as more valuable for heat-risk management. Third, it integrates the Functional–Expressive–Aesthetic framework with human-centered co-design to identify culturally responsive, cost-sensitive, and usability-focused design requirements for future prototype development.

## 2 Related work

### 2.1 Smart garments and wearable technologies for athletes and connected health

Smart clothing (e-textiles) usually comprises sensors of both physiological and environmental parameters, on-garment feedback mechanisms (such as LED indicators or textile-based displays), as well as wireless connectivity capabilities (through Bluetooth connections to smartphone apps) (16, 17). Within the realm of sports, such solutions are suitable for the purpose of monitoring athletes' heart rates and physical activity patterns. Smart clothing may offer on-the-spot notifications about athletes' current state, as well as serve as an instrument that helps evaluate the risks associated with their activities by sending action messages to athletes and their coaches (18). With respect to endurance athletes working out in hot environments, users' needs and preferences go beyond mere monitoring of physical performance. The comfort, proper fit, usability (e.g., simple, localized notifications), cost-effectiveness, and durability of smart clothing matter greatly when it comes to wearing digitally enabled sportswear (13).

### 2.2 Smart wearable technology and heat illness treatments

Heat illness results from metabolic heat production, as well as environmental stresses that exceed the ability of the body to shed heat through conductance, convective heat transfer, radiation, and evaporative cooling processes (1). Physiological sensors (such as heart rate, skin temperature, sweat loss) and environmental heat-stress indices (e.g., Wet-Bulb Globe Temperature, WBGT can be used to give warning about impending heat stress and dehydration problems (6). As most current solutions simply monitor and warn, athletes exercising in the tropical environment often require active interventions. Recently developed cooling clothing technologies using air, fluid, Peltier technology, passive phase change material cooling or radiant cooling fabrics and hybrid approaches, such as harnessing natural wind flow coupled with mechanical cooling techniques can provide personalized zonal cooling that can be automated through sensor activation (when certain temperature and HR thresholds are exceeded) (13). Sport-specific design considerations include personal body mapping and personalized control to optimize cooling vs. muscle warming. Financial constraints, especially in developing countries, influence the viability of technology.

Cross-domain thermal-comfort research also provides useful guidance for smart sportswear design. Although vehicle occupants and endurance cyclists differ substantially in exertion level, posture, sweating, exposure, and safety requirements, recent work on driver thermal comfort shows the value of adaptive thermal regulation, biometric monitoring, intelligent environmental sensing, user-controlled comfort, and energy-efficient cooling systems in mobile heat-exposed settings (19). These insights support the present study's emphasis on sensor-informed localized cooling and simplified feedback, while also reinforcing the need to adapt such systems to the washability, weight, durability, and movement demands of cycling garments.

Emergency heat illness treatments consist of rapid cooling, fluid replacement, and physiological support (20). Early detection can reduce the risk of serious health impacts and improve the effectiveness of these treatments. Researchers have explored smart wearable technology to design and develop product concepts that detect heat-illness signs early and alert users. One such concept is monitoring body hydration to understand the risk of dehydration (21). Dehydration is a common symptom of heat illness, particularly when exercising in hot, dry climates. It is evident that under hot and dry conditions, 98% of the body heat dissipates through evaporation, causing dehydration (1). Early detection of this condition enables faster and more effective treatment.

Researchers have also explored Physiological Monitoring Systems (PMS) that monitor multiple physiological parameters such as heart rate, respiration rate, skin temperature, body heat flow, and core body temperature of individuals in real-time to alert the wearer or remote personnel on potential heat illness risk (6, 22). Some PMS are available commercially, such as Polar H10, Garmin HRM-Run, and Smart Patch (6). Pham, Yeap (23) designed a wearable device that explored the relationship between an individual's behavior and physiological factors to determine the personal heat risk. Yi, Chan (24) designed a heat stress warning system that focused on workers exposed to hot and humid climatic conditions. This system captures four types of data to determine the heat risk. These include: (a) immediate WBGT readings of the construction site, (b) time spent and nature of work performed by workers, (c) individual worker information, such as their age, height, weight, consumption of alcohol, and whether they smoke, and (d) immediate heart rate of the workers. By considering the estimated risk level, the system will suggest mitigation measures for reducing heat stress either through symptomatic treatment or notifying the safety, medical or supervisor personnel.

Thus, smart and sensor technologies are widely employed in the health and medical domains to detect heat illness risks. However, most of these concepts only focus on alerting the individual/remote person about the risk of heat illness rather than delivering treatment to the user. Some researchers have combined physiological monitoring with smart cooling (rapid cooling is a heat illness treatment) to design auto-cooling technologies (25), shifting PMS systems to the next level. In addition, smart self-perspiration garments can emit oozing water (26), and these concepts can be further developed as body hydrating technologies (fluid replacement is one of the treatments for heat illness). However, the suitability of these auto-cooling and/or self-perspiration concepts for heat illness treatment or for athletes during competitive sports has not been adequately explored.

In addition, we are unaware of any research exploring the medical/sport science considerations of smart cooling design to determine the optimal physiological conditions to activate smart cooling in the outdoor endurance sports domain. Moreover, such considerations will also assist in determining the most suitable body zones for smart cooling function and the optimal cooling level needed for the athlete considering athletes' heat-sensitive body zones, the heat exposure angle during outdoor sports (like cycling), wind conditions, outdoor temperature, variation in the temperature during endurance cycling, and psychological comfort requirements of the athlete. Such research contributes to improving the health of athletes by avoiding heat health impacts and offering optimal cooling comfort, ultimately improving sports performance. Therefore, we conducted this study to explore the design considerations for smart sportswear for cycling athletes living in a developing country, and to co-design smart sportswear for these athletes.

### 2.3 Culture and co-designing digital health sportswear

Culture shapes technology acceptance, aesthetics, and perceived value. Culture creates national, ethnic, geographic, and socioeconomic boundaries (27). Culture and design are interrelated disciplines that play a key role in civilization (28). The evolution of objects has triggered human evolution, which has been shaped by cultural characteristics. As further explained in the literature, culture defines human behavior and acts as a filter that determines the suitability of a new product concept for the community (29). According to Gaver (29), failure to consider cultural values in product design can result in consumer rejection of product concepts.

Researchers have explored cultural considerations in product design. One study examined faith-based design: how religion and beliefs that exist in society could influence the design of technology-embedded product concepts (30). As explained in this study, the textures, shapes, and functions of technology-embedded product concepts are molded by target consumers' religious beliefs to enhance user-perceived value. Another study explored cross-cultural functional and aesthetic design considerations for denim jeans (31). According to the results of this study, conformity to product design is an important criterion that determines the product purchase decisions. These consumers preferred moderate design features over the latest fashionable jeans to minimize the risk of disapproval from the community. Chinese consumers prioritized the functional features and quality of denim jeans rather than aesthetics, which would make them stand out in society.

In contrast, Canadian consumers prioritized aesthetic features and preferred unique aesthetics that made them stand out in society (31). Another study explored the clothing preferences of Taiwanese female Baby Boomers and found that modesty and appropriateness to society/age are important clothing design considerations (32). Stewart and Janovicek (33) explored historical female dress in France. According to this study, historical French women preferred clothing that could make them slim, not as a fashion preference, but to conform with the ideal female figure accepted by society.

Thus, culture is crucial in determining product design considerations. Researchers have used a user-centric approach to co-design smart sportswear concepts to enhance the health and well-being of individuals (34). However, most existing studies on smart garments have considered the needs of athletes in developed countries (34, 35), overlooking the requirements of athletes living in developing countries. Co-design techniques help identify not only health functionality requirements but also aesthetic requirements (such as use of the Sinhala user interface and icons for instructions) and local cycling dangers (nighttime visibility and fall prevention). In cases where resources are limited, co-design techniques can assist in determining which features to keep that are essential for maintaining health (cooling system, hydration reminder, emergency communication) and low complexity interfaces.

The aim of the study is to understand the requirements for smart garments that can enhance the wellness of athletes. Therefore, we adopted a co-design approach to answer the following questions.

RQ1: What are the considerations for developing smart sportswear to support cooling comfort for endurance cycling athletes?

RQ2: What are the prioritized design attributes of smart cooling sportswear that cycling athletes should accept?

In this study, we have demonstrated how co-design as a process helps elicit and integrate specific cultural, contextual and technological factors to design a smart sportswear.

### 2.4 Theoretical framework linking apparel design and digital health

The Functional–Expressive–Aesthetic (FEA) model offers a clear lens for translating user needs into apparel requirements (comfort, protection, fit; self-image; appearance). For digital health sportswear, FEA should be paired with human centered design (HCD) and co design methods to ensure that smart functions (monitoring, alerts, compression/recovery, communication) are usable, safe, and culturally responsive. Practically, this means: (1) functional—zonal cooling mapped to sweat/heat exposure; reliable sensing and simple alerts; (2) expressive—UI language/localization and on garment visual feedback (e.g., recovery level colors); (3) aesthetic—visibility features and materials appropriate for tropical climates (light colors, perforation). Integrating FEA with HCD thus bridges design quality and digital health impact.

Designing functional apparel that is not only functional but also attractive was a challenge for apparel designers. Lamb and Kallal (36) addressed this concern by proposing an apparel design framework that can be adhered to by designers of functional clothing. Since simplicity was identified as a necessity by people in this rapidly evolving world, the Lamb and Kallal apparel design framework was based on six steps that can easily be adopted even by novice designers. This includes problem recognition [the understanding and analysis of the requirements of the user for his functional, expressive, and aesthetic (FEA) needs], initial idea generation, design iteration, prototyping, evaluation, and implementation. Also, the design process suggests the use of the functional, expressive, and aesthetic (FEA) design approach while recognizing and evaluating problems during the design process. Thus, the model explores design requirements by studying the functional, expressive, and aesthetic needs of the user (36).

Functional needs define the utility/benefits of the product and consist of functions related to user comfort, protection, fit, and ease of movement. Certain apparel is defined based on these functional attributes and is known as functional apparel (36). Sportswear is one such functional apparel category. Prior studies evaluated the functional needs of various user groups, and one such study identified free movement, comfort, fit, and sun protection as the key functional requirements of mature female golf players (37). Dickson and Pollack (38) identified fit and comfort as key aspects, which determine user satisfaction for sportswear and improve sports performance. Another study identified overheated or insufficiently padded cycling wear as key functional concerns for female cyclists (39). One user study identified thermal physiology and bio-mechanics as important considerations in defining the functional requirements of professional cycling wear (40). Chae, Black (41) identified comfort and fit as the most significant functional requirements for female tennis players. Regarding sailing apparel, one study identified ease of movement in a confined space as an important functional requirement (42). These functional requirements, identified by previous studies, heavily depend on user characteristics and the intended use of the apparel.

The flexibility of the apparel design framework proposed by Lamb and Kallal (36) enables researchers to apply this framework in combination with other design process models and theories (43). Also, this framework allows designers to simplify complex user needs to define design considerations for functional apparel. Considering these, the study used the FEA model proposed by Lamb and Kallal (36) to understand design considerations for cooling sportswear.

## 3 Methodology

This study employed a human-centered co-design approach to identify and prioritize smart sportswear features that integrate digital health functions for heat illness prevention in tropical, low-resource settings. User engagement and collaborative development have been identified in the literature as effective approaches to introducing the technology-enabled concept to developing countries such as Sri Lanka (44). The interactive nature of co-design workshops, which allows participants to express their ideas and perceptions in an informal and creative setting, plays a key role in facilitating user engagement in the prototype design phase. Several researchers have used co-design workshops to design products and applications for developing countries, such as Sri Lanka (45, 46), thus demonstrating the successful outcome of co-design workshops in developing countries. Hence, we employed co-design workshops to design sportswear concepts for Sri Lankan athletes.

### 3.1 Ethical approval and consent

Ethical approval (#2018/426) was obtained from the Human Research Ethics Committee of our university. Written informed consent was obtained from all participants before participation and after explaining the scope of each user study in both English and Sinhalese (the native language of the target groups). Participants were also asked to provide consent to audio record the interviews and discussions. They were informed that participation in this project was voluntary and that they could withdraw consent to participate at any time. If a participant wished to withdraw from the study, his/her records were deleted, and those records were not used for the research. We offered AUD 20 gift vouchers to study participants as an appreciation for their efforts.

### 3.2 Participants and sampling

Convenience sampling was used to recruit participants. The participant inclusion criteria were as follows: a) age of 20–45 years and b) endurance cyclists who participated in at least 70 km or more extended cycling events within the last six months. Participants were recruited with the help of Sri Lankan sports organizations and cycling clubs. In addition, the first author visited the 3-day endurance cycling race in Sri Lanka to facilitate further recruitment and familiarize themselves with the riders. We recruited 20 semi-professional endurance cyclists (19 male, 1 female) aged 20–45 years who had completed at least one 70 km cycling event in the past six months. In this study, “semi-professional” refers to cyclists who regularly participated in organized endurance cycling events or races, were affiliated with sports organizations or cycling clubs, and had recent experience completing events of at least 70 km but were not necessarily full-time salaried professional athletes. Because recruitment was conducted through clubs, sports organizations, and an endurance cycling event, the sample may represent more active and competitive cyclists rather than the broader population of recreational Sri Lankan cyclists.

### 3.3 Co-Design workshops

The author employed co-design workshops to facilitate user engagement in the prototype design phase and to understand consumer perception to define the input requirements for initial prototyping. The co-design study consisted of four idea generation workshops (*n* = 5, per session) and one screening workshop conducted with 20 Sri Lankan endurance cycling athletes (19 male and one female). All the co-design workshops were held at the premises of a sports organization in Colombo, Sri Lanka.

The co-design workshops were conducted on the premises of one of the Sri Lankan sports organizations in Colombo. All of the study activities were conducted in the Sinhala (the native language of the participants) language and were audio-recorded for further analysis. The participants were provided with the necessary toolkits (idea-generation: papers, stationery, and screening: sketches, pens, sticky notes). The first author facilitated all co-design sessions.

#### 3.3.1 Phase 1: idea-generation workshops

We conducted four brainstorming workshops (five participants per session) with 20 participants. See Table 1 for an overview of the co-design activities. Each session lasted for 2.5 h. During the sessions, the first author conducted an introductory presentation explaining the objectives of the project and the procedure (10 min). Afterwards, the participants were allowed to introduce themselves (10 min). Subsequently, we requested that the participants express their endurance sportswear expectations and concerns (40 min). Subsequently, we conducted an icebreaker activity by requesting the participants to express their experience as a cycling athlete (15 min) to create an informal and friendly environment before the idea-generation session. They could use only three words, which allowed them to explore their creativity.

**Table 1:** Co-design activities.

Design Workshops	Activities	Participants	Outcome
Five sessions in total. Each session was 2.5 h	Introductory presentation (10 min)	20 participants (5 in each session)	Cycling sportswear concerns and 19 Smart sportswear design ideas
	Introducing team members (10 min)	19 males and one female; Semi-professional cycling athletes engage in 70 km and above competitive cycling races in Sri Lanka	Prioritised smart garment design suggestions
	Group discussion (40 min)		
	Ice-breaker activity (15 min)		
	Video demonstration (15 min)		
	Idea-generation/sketching (1-hour)		
	Screening workshop (30 min)		

**Figure 1:**
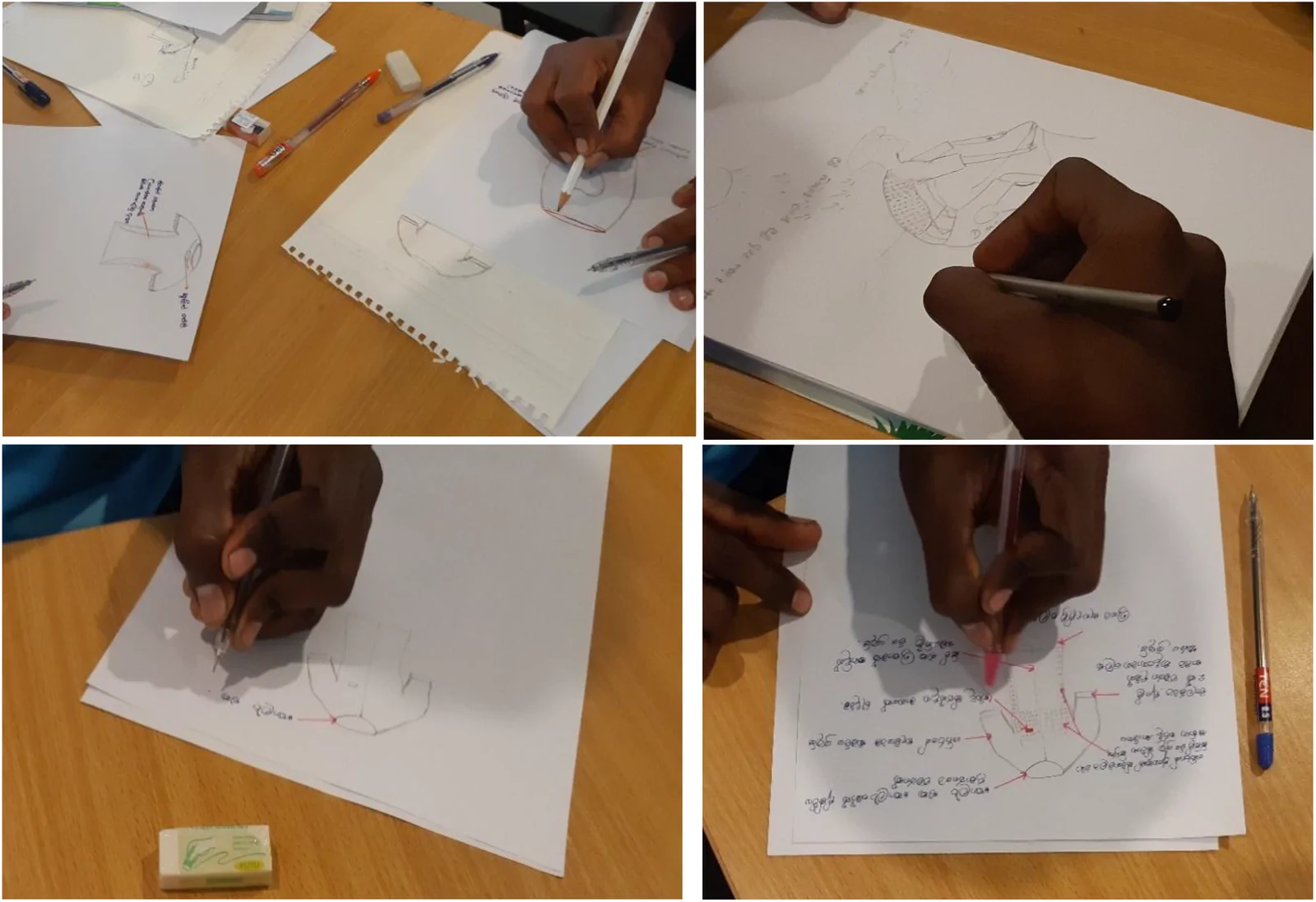
Participants generating smart sportswear design ideas.

After this activity, the researcher presented videos of popular smart garment/clothing concepts [for example LUMO run (47), Athos garment (48), and Nadi X smart yoga pants (49)] to the participants (15 min) to make them aware of existing smart garment technologies. Finally, we requested the participants to express design ideas for smart sportswear that could improve their well-being (1 h) (Refer to Figure 1). During these workshops, participants used tools such as whiteboards, post-it notes, pens, pencils, and paper to express ideas. Finally, they discussed the suggested designs with their fellow group members. Finally, the first author concluded the session with an informal discussion, allowing volunteer participants to elaborate their sketches verbally.

To reduce the influence of dominant voices during group activities, participants first generated sketches and written ideas individually before discussing concepts with the group. Nevertheless, because the workshops involved group discussion and consensus building, the possibility that more vocal participants influenced later prioritization cannot be fully excluded.

#### 3.3.2 Phase 2: screening workshop

The workshop was conducted after completing all four brainstorming workshops. The screening workshop was conducted one week after the brainstorming workshop and lasted for 30 min. During the screening workshop, we allowed the participants to evaluate the 19 design concepts proposed during idea generation workshops and to prioritize the most important smart functions for sportswear based on their importance and preferences. Participants used sticky notes to add additional features (if needed) and evaluate the design concepts. Finally, they ranked features based on health impact, usability, and cost feasibility. The prioritization was conducted through group discussion, sticky-note voting, and consensus building rather than statistical ranking. Concepts that participants considered impractical, too costly, or insufficiently relevant to heat illness prevention were filtered out. We selected the final two T-shirt concepts because they represented the features most consistently supported across the screening activity: targeted cooling, airflow through perforated panels, user control, safety visibility, and cost-sensitive implementation.

### 3.4 Data collection and data analysis

We collected audio recordings of workshop sessions, written notes, and participant sketches from all workshops. Voting tallies were also recorded to identify the most frequently suggested cooling strategies for subsequent screening and refinement. All data were translated into English when finalizing the transcripts. The first author, whose first language was Sinhala, and a graduate who completed her higher studies in English conducted the translations. However, the translations were verified by another Sri Lankan graduate, whose first language was Sinhala and who conducted higher studies in English. The author assigned pseudonyms to the participants (P1–P20) to ensure confidentiality.

Finally, we conducted a thematic analysis (50) of the translated transcripts and design sketches using the NVivo 12 software. A thematic analysis, consisting of both inductive and deductive analysis, was used to process the outcomes of the idea generation workshops. Initially, the authors read the transcripts and referred to the design sketches thoroughly to be familiar with the results. Afterwards, the author followed the functional, expressive and aesthetic (FEA) model (Lamb & Kallal, 1992; Orzada & Kallal, 2016; Steinhardt, 2010) to analyze the transcripts. Data were analyzed using Braun and Clarke's six-phase thematic analysis process , comprising data familiarization, initial coding, theme development, theme review, theme definition and naming, and the production of the analytical account. Initial codes were generated inductively from participant statements, sketches, and workshop notes, while the FEA model provided a deductive structure for organizing codes into functional, expressive, and aesthetic requirements. The first author conducted the initial coding, and another researcher reviewed the coding structure and theme development. Ambiguities and disagreements were resolved through discussion and, where necessary, by returning to the original Sinhala transcripts and workshop notes. Themes were linked to digital health objectives (e.g., usability of localized UI, simplified alerts, integration of cooling and monitoring).

Although the workshops produced several design ideas, these were not treated as separate final requirements. Instead, sketches and discussion data were clustered into recurring categories and themes. Common ground was identified through recurrence across participant sketches, verbal explanations, sticky-note outputs, and screening workshop prioritization. Thus, the sample size was considered appropriate for exploratory co-design because the aim was to identify repeated design concerns and priority directions rather than to estimate population-level prevalence.

We followed several strategies to improve the quality, integrity, and trustworthiness of the qualitative data. All user studies were conducted in Sinhalese (the native language of the target groups) to capture the participants' feedback accurately. After each user study, the author discussed key findings with the participants and obtained their consent. The first author translated the participants' feedback (audio and written) into English. Another researcher verified these English transcripts. Both researchers were native Sinhalese speakers and international graduates. The first author independently conducted qualitative data analysis and verified it with another qualified researcher. Trustworthiness was further supported through the use of multiple data sources, including audio recordings, written notes, sketches, sticky-note outputs, and participant quotations. These materials enabled triangulation across verbal explanations and visual design artefacts.

## 4 Results: design workshops

The co-design workshops generated 19 design concepts for smart cycling sportswear (see Supplementary material: Table A). In summary, the 31 sketches present 12 unique cooling concepts that are thoughtfully integrated into garments, focusing on functionality, comfort, and adaptability to different needs (See Table 2). Analysis revealed three dominant themes: functional requirements, usability and cultural considerations, and cost constraints.

**Table 2:** Co-designed garment types, cooling concepts (*n* = 12), design features, and concept IDs.

Garment Type	Cooling Concepts (*n* = 12)	Design Features	Concept IDs
T-shirt	Wind cooling (Perforated material insertions) ​	Perforated material for airflow. ​	4, 6, 8, 16, 17, 20, 23, 28, 30
T-shirt	Wind cooling (Evaporative/perforated material) ​	Perforated material, evaporative cooling. ​	1, 21 ​
T-shirt	Wind cooling (Perforated material insertions), Cool gel pack ​	Perforated material, cool gel pack for targeted cooling. ​	10, 12, 16, 26
T-shirt	Wind cooling (Perforated material insertions), Cooling material ​	Perforated material, cooling material for temperature regulation. ​	10, 12
T-shirt	Wind cooling (Perforated material insertions), Cooling pack ​	Perforated material, cooling pack for temperature regulation. ​	20, 21
T-shirt	Water cooling concept, Wind cooling ​	Water cooling mechanism, wind cooling. ​	25
Shorts	Wind cooling (Perforated material insertions), Cool gel pack ​	Perforated material, cool gel pack for targeted cooling. ​	5, 18, 22, 31
Shorts	Cool gel pack ​	Cool gel pack for targeted cooling. ​	7, 9, 15, 29
Shorts	Wind cooling (Perforated material insertions), Cooling pad ​	Perforated material, cooling pad for temperature regulation. ​	19
Shorts	Cooling material, Cool gel pack ​	Cooling material, cool gel pack for temperature regulation. ​	11
Sleeve	Wind cooling (Holes to remove sweat), Cooling material, Sweat absorbing material ​	Sweat-absorbing material, perforated holes for ventilation. ​	13
Pant	Wind cooling (Holes to remove sweat), Cooling material, Sweat absorbing material ​	Sweat-absorbing material, perforated holes for ventilation. ​	14

The distribution of concepts in Table 2 is presented to show the spread of participant-generated ideas and recurring design directions. These counts should be interpreted descriptively, as indicators of concept recurrence within the workshops, rather than as statistical frequencies representing the broader Sri Lankan cycling population.

### 4.1 Cooling comfort as priority

During the workshops, all participants identified cooling comfort as an essential requirement for cycling sportswear owing to the hot climate conditions in Sri Lanka. Moreover, the high-humidity conditions in Sri Lanka restrict sweat evaporation from the body, further increasing heat discomfort.

“Wind can increase cooling from evaporation because the speed of the air/wind increases evaporation”—P8.

“When we increase cycling speed, wind flow increases cooling comfort. We would like this type of wind cooling. It's comfortable for us”—P9.

Participants prioritized cooling strategies that emphasized a) airflow-based solutions such as natural wind cooling and perforated panels, b) targeted relief through localized cooling packs and c) user-controlled smart cooling, with a clear preference for hybrid designs.

The participants liked the natural wind cooling effect that could improve evaporative cooling in humid tropical conditions, and the existing cycling wear used open construction and perforated materials to promote evaporative cooling and wind cooling. Examples include laser-perforated microfiber jerseys, mesh-knit polyester/elastane panels, and lightweight 3D spacer fabrics that promote airflow while maintaining structure. Most of the design ideas suggested by the participants utilized natural wind-cooling effects to integrate cooling comfort into sportswear with the help of perforated materials. One recurring concept was an open-structure, wind-circulation jersey design, in which participants proposed front openings and/or chest perforations to channel airflow across the torso to enhance sweat evaporation and perceived cooling in humid conditions.

“The top chest has holes, which allow air to circulate inside the body to cool the body continuously”—P20.

Across these wind-cooling concepts, participants also described pairing airflow-based cooling with a minimal sensing configuration (e.g., chest-located heart rate/skin temperature) to enable simple heat-risk cues and optional cooling actions, extending the garment from passive comfort enhancement toward an actionable digital health intervention.

**Figure 2:**
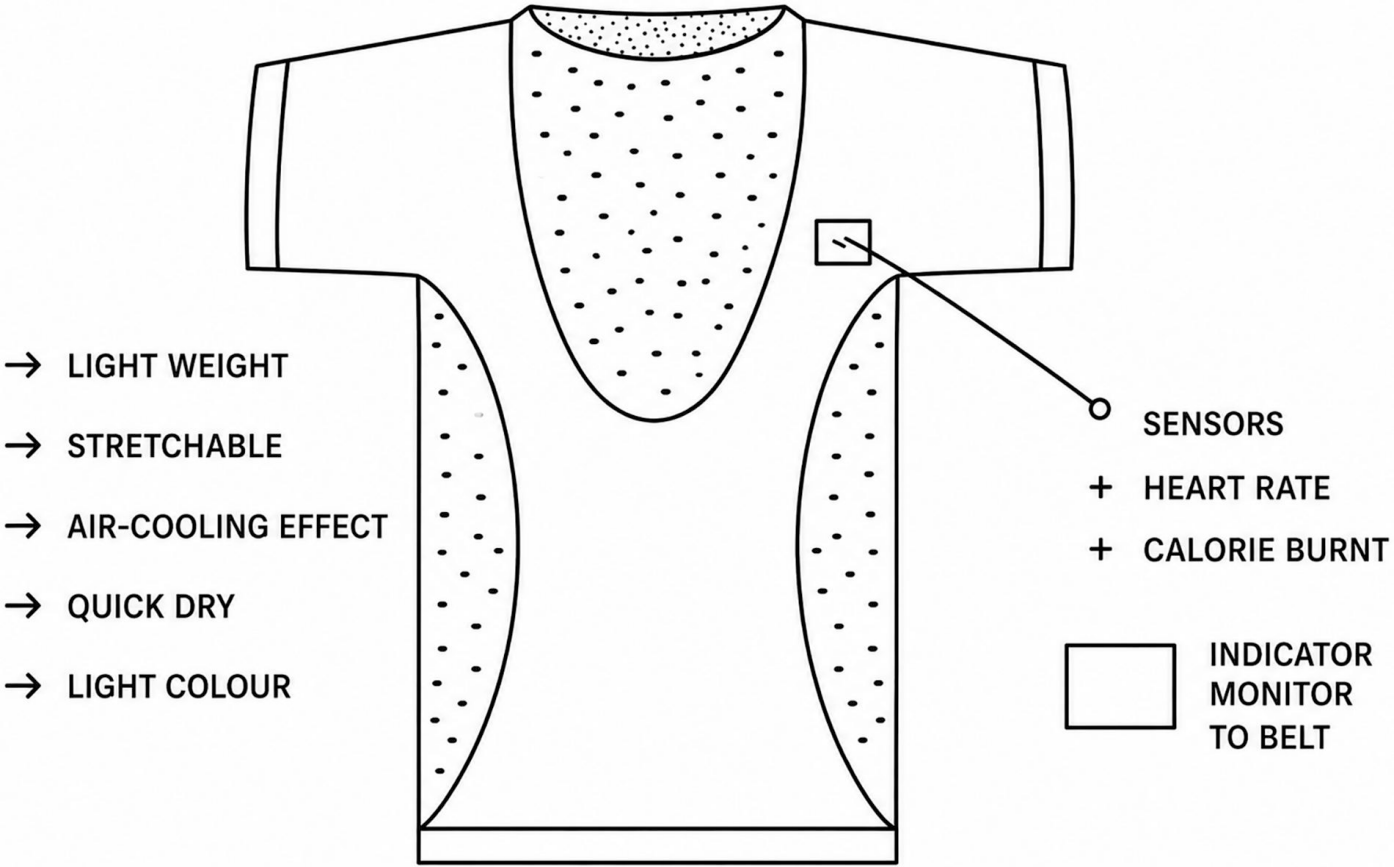
Proposed wind cooling design idea with perforated upper-back and underarm panels.

Another participant proposed a wind-cooling concept that uses perforated materials strategically placed in high-sweat zones particularly the underarm and upper-back regions to promote airflow and evaporative cooling without adding bulk (See Figure 2). Alongside this ventilation-first approach, participants also suggested minimal, alert-only sensor cues (rather than continuous dashboards), layered onto the garment to support heat-risk awareness while keeping complexity and cost low.

Across these early concepts, participants described pairing ventilation-focused fabric architectures (e.g., open-front channels; perforated underarm/upper-back panels) with a minimal sensor stack (HR and skin temperature) to enable on-garment color cues when thermal strain exceeds thresholds; powered cooling was considered optional and applied only where indicated (e.g., upper back).

Participants also developed a wind-cooling garment concept incorporating a back-mounted water reservoir intended to provide localized conductive cooling while riding. In this concept, a cool-water compartment sits against the upper back to absorb body heat, and the stored water can also serve a practical dual use as drinking water. Participants further described using a front panel made from “high-tech” temperature-regulating textiles to help maintain cooling performance by supporting airflow and heat transfer around the torso and reservoir. By “high-tech material” we mean textiles developed for temperature regulation, such as phase change material laminates used for transient cooling, radiative cooling fabrics with higher thermal emissivity, and highly permeable knit textiles with wicking treatments to maintain evaporative cooling.

Front *z*ippers with openings were another suggestion provided by the participants to promote wind cooling. Some researchers have suggested sportswear with a zipper to allow wind cooling and improve wearability. More broadly, participants proposed combined concepts that integrate wind-cooling features (perforations/openings), a localized cooling pack at the upper back, and basic sensing (e.g., HR/temperature) to support simplified hydration/cooling prompts.

Even though the participants preferred the wind cooling effect, as they further stated, the uncontrollable nature of wind cooling could affect their cooling comfort, and they favored having advanced/smart cooling technology that worked together with wind cooling to enhance their cooling comfort.

“Wind speed is not controllable, and sometimes we don't have enough wind flow. Therefore, wind cooling alone was insufficient. A cooling technology that provides consistent cooling should be developed. If a cooling sportswear can be designed with advanced cooling that utilises natural, free wind flow, then a cost-effective, advanced cooling sportswear can be designed”– P9.

Cooling packs were the second most suggested concept by the participants for obtaining the required cooling comfort. Existing cycling wear does not contain cooling packs; yet the participants have seen cycling garments with cooling packs worn by international cyclists. They believed that cooling packs could provide high cooling in hot and humid conditions; however, the participants stated that cooling packs could also increase the weight and bulkiness of the garment. Even though the cooling packs are generally made from gel, some participants preferred a lightweight cooling pack made from pre-cooled, high-absorbency fabrics rather than using comparatively heavy gel cooling packs. In their design ideas, the participants included cooling packs for the body areas that they considered the most heat-sensitive.

Inspired by some of the cooling cycling wear they saw online, some participants suggested using cooling materials/fabrics in garments with an inbuilt cooling function that could enhance cooling comfort. The use of light-colored materials was another suggestion to improve light reflection and cooling. However, some participants had concerns regarding the cooling levels achieved only by the materials.

“Fabrics can be improved to enhance sweat removal to improve cooling slightly. In addition, using light-colored fabrics slightly reduces sunlight absorption. However, I do not think thin fabric alone can provide sufficient cooling for cycling conducted in hot climates because they cannot store cooling or create cooling effects like AC to give cooling in this hot climate”—P12.

The participants suggested exploring advanced/smart cooling concepts that can offer a forced cooling effect regardless of environmental conditions, such as AC/fan. Regardless of the suggestions to increase cooling comfort, the participants stated that having high cooling for a longer duration might cause discomfort to athletes. In addition, as elaborated by them, when riding in low or moderately hot climate conditions, such as in the upcountry, wearing a cooling cycling garment can be very uncomfortable. Considering these concerns, the participants suggested integrating personalized cooling control functions into the cooling cycling garment. According to them, an advanced cooling technology offering user-controllable functions would fulfil this requirement. Few participants suggested exploring the auto-cooling concept to provide personalized cooling comfort.

### 4.2 Cooling zones

Participants identified neck and upper back as the most heat-sensitive zone, followed by underarm and chest. These zones were prioritized for cooling features to avoid interfering with muscle warm-up. The participants incorporated cooling functionality into some regions of the sports garment that they considered the most heat-sensitive.

“We designed this jersey to have a cool gel pack around the neck, which goes up to the shoulder. Even during a race, when we get tired, we put water in this area to cool the body without affecting its temperature” (P11).

As elaborated by the participant, the behind-the-neck/upper back (on and slightly below the neck in the back) was considered the most heat-sensitive body zone. Cooling this body area enables athletes to perform without causing discomfort. As further expressed by the participants, this is also the area most exposed to direct sunlight during riding; hence, the participants preferred to cool this area of the body to eliminate heat discomfort from external sunlight. Furthermore, because the back-neck/upper-back areas of the body have few active body muscles, by cooling these zones, participants would be able to cool the body without affecting the internal body warm-up required for the activity. Another aspect stated by the participants was that the temperature of the entire body could be controlled by regulating the temperature in the back, neck, and upper back areas.

Some participants considered the cycling pad area, underarm, chest, shoulders (T-shirt and bib straps), sleeves, and back as important body zones subjected to heat discomfort during riding. A few participants suggested integrating cooling into the whole body rather than focusing only on selected body zones; nevertheless, the body warming up of active muscles needed to be considered to avoid an impact on race performance. Furthermore, the participants identified the upper back, chest, and underarms as the main sweating areas that promote evaporative heat removal. Given the gender imbalance in the sample, these body-zone and fit findings should be interpreted as preliminary; future prototype studies should include gender-specific fit and cooling-zone mapping.

### 4.3 Smart functions

Beyond cooling, participants suggested a) physiological monitoring (heart rate, hydration, temperature), b) compression for recovery, and c) emergency communication features. However, monitoring-only solutions were considered less valuable than cooling interventions, reflecting a preference for actionable health features (See Table 3).

**Table 3:** Smart functions for cooling cycling sportswear.

Smart Functions	Design Considerations
Smart Monitoring	Smart Monitoring
	Suggested biological parameter monitoring technologies for smart cycling sportswear.
	Heart rate, burnt calories, cramp alert, speed, temperature (air and body), hydration. Suggested performance and physical monitoring technologies for smart cycling sportswear.
	Posture, speed, effort applied, speed and distance of the competitor team, detect/identify difficulties in the road ahead.
	They preferred having smart cooling technology that can minimise the occurrence of heat illness rather than having smart monitoring technologies that can only alert them of heat illness risk.
Smart Compression	Design sportswear with smart compression/recovery functions to ensure fast recovery during endurance races lasting several days continuously.
Communication	Microphones embedded in the sportswear to ensure communication among team members while riding. This can also support communication for emergency health services.

#### 4.3.1 Smart monitoring

Heart rate monitoring was the key function suggested by the participants, as some of them had already used heart rate monitoring technology.

“Measuring heart rate helps to prevent accidents that can occur due to heat”—P17.

As mentioned by the participant, heart rate measurement warns them of potential health threats which could arise due to excessive heat. The participants further mentioned that the significance of heart rate (HR) monitoring is high in tropical countries such as Sri Lanka, where the risk of heat illness is higher because an increase in heart rate is an indicator of heat illness. Body/environmental temperature monitoring is another bio-monitoring function that has been suggested and is important for the Sri Lankan climate. Knowing their body/environment temperature allows cyclists to be alert to possible heat illness situations that might arise. Burnt calorie monitoring was also considered an important function, and body hydration level monitoring was another suggested smart function. As mentioned by the participants, owing to high-temperature environmental conditions, they experienced elevated levels of sweating that could lead to dehydration. Hence, if the garment could alert them when their body hydration level fell below level a safe level, they could drink adequate water to avoid dehydration during the races. One participant suggested including a muscle cramp detection mechanism for cycling shorts.

“The garment should have a smart technology to alert the rider about cramps so we can take precautions to avoid the occurrence of cramps”—P18.

As further elaborated by the participants, the occurrence of cramps could significantly inhibit their performance. Therefore, if the cycling garment alerts the wearer to cramps in advance, they could take precautions. Other functions have been suggested for detecting speed, revolutions per minute (RPM), and the effort applied to smart garments. Some participants came up with ideas for advanced smart monitoring functions, such as detecting posture, detecting the speed and distance of the competitor team, and detecting/identifying difficulties in the road ahead. Participants also suggested using sensors to incorporate smart biomonitoring functions into the garments.

“The chest should have a sensor to measure the heart rate”—P16.

Some participants preferred a mechanism or technology that gave instructions to the rider based on measured parameters, rather than merely displaying the values.

This preference should not be interpreted as a rejection of physiological monitoring. Several participants were familiar with heart-rate monitoring and recognized its value for heat-risk awareness. However, monitoring was perceived as more useful when it generated simple instructions or triggered actionable support, such as hydration prompts or cooling activation, rather than when it only displayed numerical data.

“The top has a smart system to measure heartbeat, temperature, the water level in the body and to provide instructions to the rider when water intake is required and how water intake should be conducted to avoid dehydration”—P19.

Because no functional prototype was tested in this study, we do not propose validated activation thresholds. However, future sensor-triggered cooling and hydration alerts should be developed and tested using a combination of physiological, behavioral, and environmental indicators, such as skin temperature, heart-rate deviation from individual baseline, elapsed riding time, perceived exertion, sweat-loss or hydration-risk indicators, ambient temperature, humidity, and Wet-Bulb Globe Temperature. Cooling activation should therefore be treated as an empirically testable design rule rather than a fixed clinical recommendation.

Participants were concerned about the instructions provided by existing smart technologies, such as Fitbit, which they had seen online. According to the participants, existing smart applications related to garments did not include Sinhala language translations. They also stated that the data displayed by these applications was too complicated for them to understand.

“The instruction and data displayed by current smart units are in English, and also, they are too complicated for us to understand”—P7.

#### 4.3.2 Compression and communication

**Figure 3:**
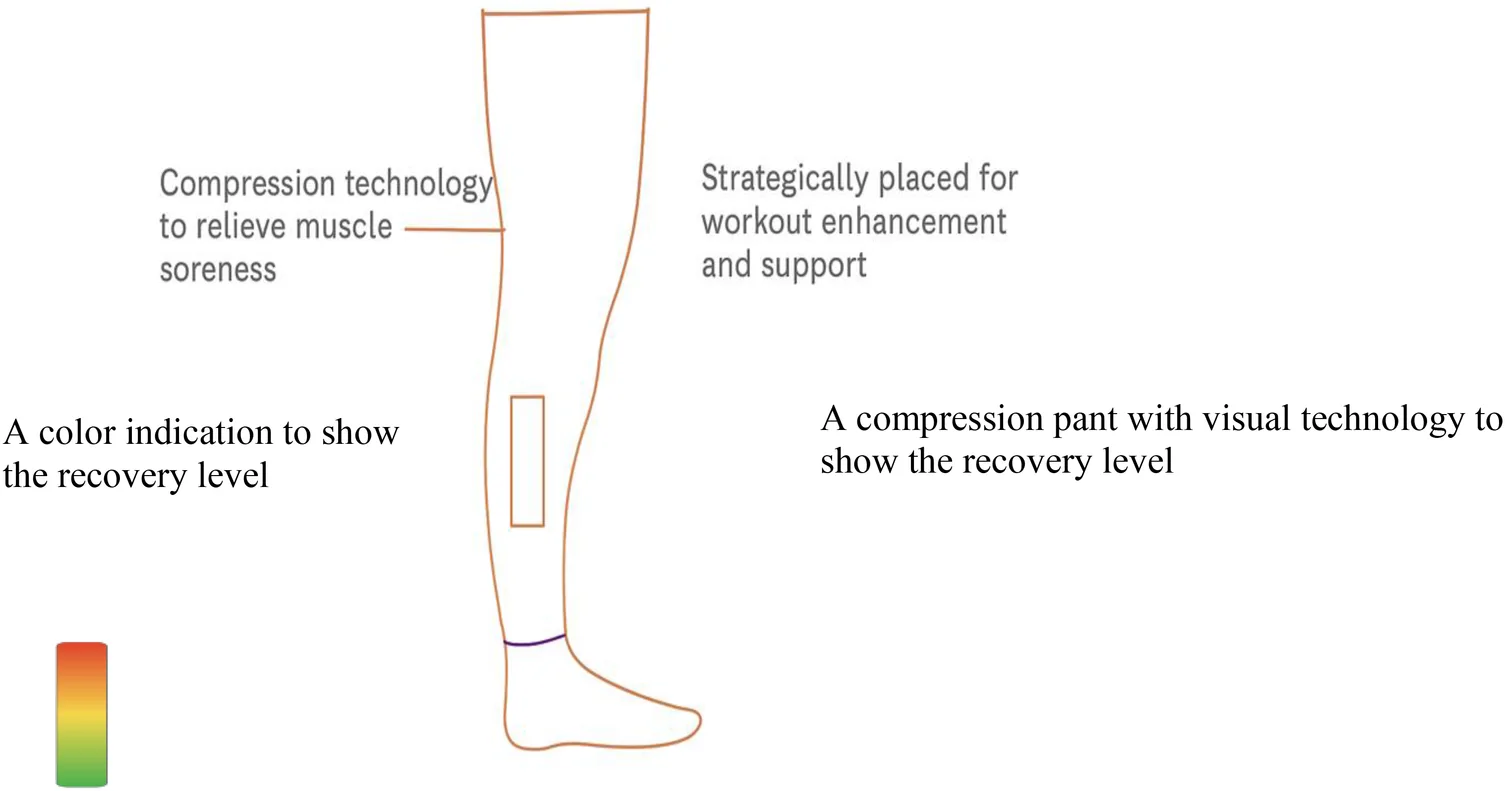
A drawing of a participant: compression pants. Participant sketch (English labels translated from Sinhala). Example implementation showing an on-garment color/LED state that maps recovery level from low (red) to high (green).

Sports compression and recovery have been suggested as smart technologies (Figure 3). They preferred sportswear that could provide post-activity compression during endurance cycling. As further indicated, endurance cycling races last for two or more days, and after completing their daily miles, they have only a few hours to rest and recover for the next day's riding. If they had post-activity compression sportswear, they would be able to recover easily the following day.

“If we race the next day, we have to use ice packs or physiotherapy to recover quickly. A recovery sportswear to do this would be very beneficial. Then the sportswear will automatically recover the body”—P11.

Some participants preferred a full-body suit to recover their body by providing intense cooling rather than a smart compression garment.

Communication is another function suggested by the participants that helps them communicate during the race and work as a team. Communicating with fellow team members and instructors during a race improves their confidence, ultimately improving their performance. In addition, this feature enables athletes to communicate their risky health situations, such as elevated heart rate, dizziness, and heat stroke, to instructors/supporters so that they can take necessary precautions to minimize the health impact. However, as further stated by the study participants, Sri Lankan race regulations do not allow microphone usage during competitive races; hence, approval needs to be obtained to design such sportswear.

#### 4.3.3 Cultural and usability insights

In addition to the sportswear functional design features explained in the previous sections, participants suggested certain other functional features that need to be considered to improve the comfort and performance of cycling wear. Concerning the T-shirt, some participants proposed easily reachable pockets so that they could access the supplements they had carried without disrupting their cycling movements. They stressed the importance of having high stability in the pocket area of the garment to avoid garment distortion that could arise due to the weight of the supplements. Another suggestion was to have additional pockets to accommodate the components related to smart functions, such as a mike (for communication).

Some participants suggested using short/long zippers in their designs to improve their wearability. As they stated, they could open the zipper to allow wind cooling if needed. However, they also mentioned concerns regarding zipper construction of existing garments.

“When bending, the zipper roll touched my body”—P2.

As mentioned by the participant, uncomfortable rough zippers in existing garments touched their bodies when they bent during cycling.

“The zipper should feel like there is no zipper, and when we bend, the zipper should not touch our stomach. If possible, the zipper should be covered by a fabric”– P1.

As stated by the participant, the zipper should be soft/flexible and thin and should not be felt by the wearer. Additionally, the participant suggested covering the zipper backside with fabric to reduce touch discomfort due to the rough zipper material and/or metal/plastic components.

Participants preferred both the with-collar and without-collar options. Generally, they preferred short sleeves on the T-shirt, yet some preferred long sleeves. Sleeve and waist-edge elastics are common features proposed to prevent the garment from rolling up during cycling. They wanted stretchable, thin, smooth-edge elastic/gel/fabric tapes for comfort. Some participants suggested using adhesive tape instead of elastic tape. Soft stretchable bands in the neck to ensure proper fitting and gel stripes inside the seams to improve comfort have also been suggested. A luminous/reflective line or illumination has also been proposed to provide night-riding visibility.

For the shorts designs, most participants suggested constructions that included bib straps to hold the shorts during cycling and recommended using soft material in the bib straps (shoulder area). Participants considered the cycling pad to be an essential component and advocated a soft, durable pad with a cooling gel. One participant suggested a novel gel pad, which could be refilled when required.

“The gel can be refilled to improve the softness of the pad. Otherwise, the softness of the gel pad can be reduced over time. The most important part of the shorts is the pad”—P16.

Some participants suggested washing a durable cushion material for the pad. Most proposed methods include stretchable, thin, and smooth elastic options (gel/fabric tapes) for the shorts' waist and shorts' the legs of their designs to avoid rolling up. Some have suggested rigid padding in the back and other suitable zones to protect athletes during accidents and falls. As stated by the participants, the existing cycling garments did not have fall protection features. Participants emphasized the importance of using stretchable, soft materials and lightweight technologies for both T-shirts and shorts. They also suggested designing a T-shirt and shorts to achieve a perfect customized body fit to minimize aerodynamic drag.

#### 4.3.4 Aesthetics

The participants stated that athletes' preferences for design features such as sleeve length, collars, and zipper lengths might vary based on individual choices for design and appearance. They mostly prefer aesthetic features linked to functional benefits. For example, they suggested integrating aesthetic features, such as illumination, into sportswear to improve visibility so that other vehicles would be aware of athletes and thus avoid accidents. In addition, participants suggested a technology to visualize smart functions, such as a color indication indicating the recovery level of a compression pant (Figure 3).

“If the compression level can be made visible, the cyclist can mentally feel it”—P12.

Some participants preferred certain aesthetic features of garments that delivered functional benefits.

“In Sri Lanka, we need light colors. These colors help to reflect heat”—P17.

#### 4.3.5 Cost considerations

Regardless of the potential benefits of smart garment technology, participants strongly mentioned that if the cost of cooling sportswear increased due to smart functions, they preferred to focus only on smart cooling because the most important requirement was cooling. As further stated, they favoured smart cooling technology in their sportswear to minimise heat illness risk rather than a biomonitoring alerting mechanism, as sometimes the monitoring details provided by the smart unit can cause errors.

“The most important thing is to have proper cooling to avoid heat illness rather than monitoring the risk level by these smart functions. We do not know how reliable the sensor data is”—P20.

They emphasised the importance of designing a smart sportswear while ensuring value for money. They also stated the significance of focusing only on essential functions when designing a sportswear cost-effectively.

Although this concept-development study did not include a formal bill of materials, the emphasis on cost feasibility indicates that future prototypes should include component-level costing and maintenance assessment. Key cost and feasibility items include temperature and heart-rate sensors, a microcontroller, battery and charging unit, cooling pack or active cooling module, conductive wiring or e-textile integration, washable pockets or sealed detachable casings, reflective trims, and Sinhala-language mobile or on-garment feedback. Practical deployment will also require attention to removable electronics, sweat resistance, washability, durability under repeated cycling posture, battery safety, charging safety, local repairability, and manufacturability through local garment producers.

### 4.4 Results: screening workshop

During the screening workshop, participants re-confirmed their most important sportswear attributes (e.g., cooling comfort, rider safety, sweat evaporation, and cost) identified during the brainstorming workshop and filtered the design features to proceed based on these attributes (See Table 4). Additionally, they identified smart cooling as the most important smart function suggested by participants. Finally, they proposed two T-shirt design concepts based on the filtered features. The screening results should therefore be understood as a qualitative prioritization of participant-perceived value rather than a quantitative effectiveness assessment.

**Table 4:** The screened design features.

Attributes	Key concepts and gaps	Suggestions and design features	Screened features
Cooling comfort	Existing cycling wear does not meet user requirements.	Suggested cooling concepts: 1. Wind-cooling, open constructions/perforated materials/zippers/water cooling designs 2. Cooling packs 3. Cooling materials/light color materials 4. Smart	
	Cooling gaps	Cooling zones:	Concepts:
	Insufficient cooling No personalized cooling comforts	Most important: Back-neck/upper-back	Perforated material Smart cooling (Design concept 1) Cooling packs (Design concept 2)
		Others: cycling pad area, underarm, chest, shoulders (T-shirt and bib straps), sleeves, back, full body	
			Cooling and Sweating zones:
			Back-neck/upper-back/underarm/chest
Sweat Evaporation	Existing cycling wear does not evaporate sweat from the body in high-humidity climate conditions.	Suggestions	
		Enhance cooling comfort to improve sweat evaporation.	
		Evaporative cooling	
		Important sweating zones	
		Underarm, chest, upper back.	
Cost	The branded sportswear available in the international market are not affordable to them. If using advanced technologies to address other requirements, cost impact needs to be considered.	Focusing only on the most essential functions. Local manufacturers	Focusing only on the most essential functions.
Rider Safety	Existing cycling wear has contrast and reflective features. There is a requirement for increased visibility considering the high road riding risk in Sri Lanka.	Visibility: Illumination/contrasts or reflective features.	Illumination/contrast or reflective features.
		Fall protection: pads.	

#### 4.4.1 Design concept 1: smart cooling in neck/upper back with user-controlled settings

**Figure 4:**
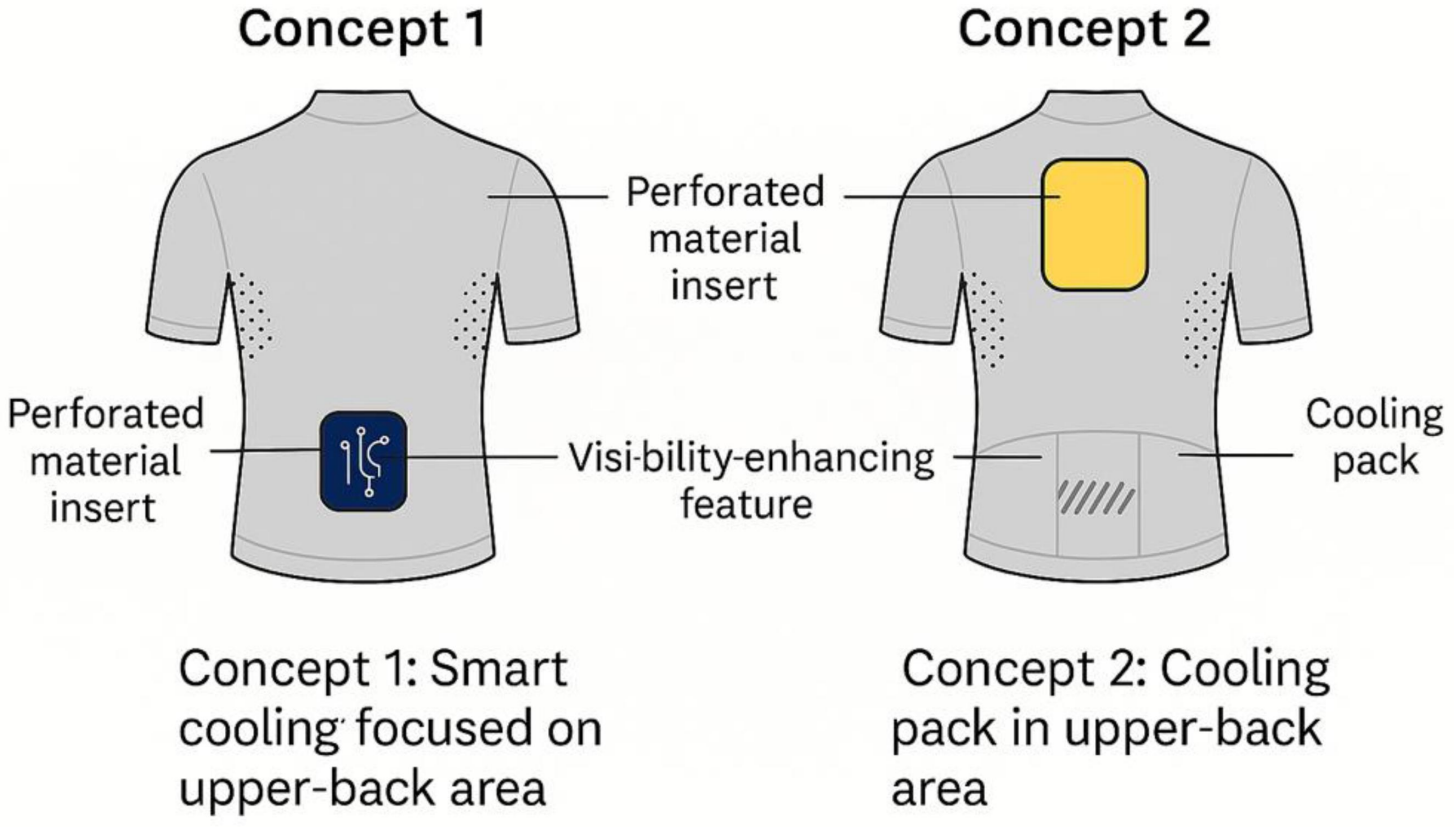
A schematic of both concepts.

Inspired by the design ideas created during the idea-generation workshop, the participants suggested using a short-sleeved non-collar T-shirt design in both the proposed design concepts. In the first concept (Figure 4: left), the participants suggested exploring the possibilities of using an advanced/smart cooling technology in the back-neck/upper-back area (the most heat-sensitive body zone) to offer a forced cooling effect in tropical climate conditions and personalized cooling (user-controllable and auto-cooling functions) that they expected.

#### 4.4.2 Design concept 2: lightweight cooling pack integrated with perforated panels for airflow

In the second concept (Figure 4: right), participants suggested incorporating a cooling pack (the second most suggested cooling concept during idea-generation workshops) into the back neck/upper back to improve cooling comfort. They suggested using pre-cooled, high-absorbency fabric in the cooling pack instead of gel packs to minimize the weight impact. In both concepts, they proposed using perforated material insertions (the most suggested cooling concept during idea generation workshops) in body sweating zones (underarm, upper back, chest) to promote heat dissipation/cooling and sweat evaporation. To achieve riding safety and after considering the suggested riding-safety-related design features, the participants requested integrating illumination/contrast or reflective visibility-enhancing features into the design options. The participants suggested focusing only on the most essential functions when developing prototypes to achieve a cost advantage. Finally, the participants screened several features for cycling shorts but stated that a T-shirt was considered the most essential garment because the upper body has most of the body sweating and heat-sensitive areas. The screened shorts featured cooling packs (between the legs) and bib straps.

## 5 Discussion

This study presents a co-design process to elicit the requirements for developing smart garments for athletes. Most of the intelligent functions suggested by our athletes have already been explored in existing studies (Belbasis & Fuss, 2018; Paiva et al., 2019); however, researchers have not explored these smart technologies considering climatic, economic, and cultural conditions related to endurance athletes living in developing countries. The study reveals that the significance of smart sportswear lies not in the mere tracking of physiological parameters but rather in culture-sensitive cooling triggered by sensors that participants perceived as useful for mitigating heat strain during physical activity. These findings remain conceptual and should not be interpreted as evidence of actual physiological benefit until prototype testing is completed.

### 5.1 Considerations for developing smart sportswear to support cooling comfort for endurance cycling athletes

The results of our co-design process indicate that cyclists prioritised cooling-focused interventions to manage thermal discomfort and perceived heat risk during endurance cycling in hot and humid tropical climates. This is reflected in the participantś consistent preference for hybrid cooling, which is the combination of natural wind flow and a smart, user-controllable system for sustaining thermal comfort when ambient airflow is not sufficient. This preference reflects both the limitations of evaporative cooling under tropical humidity and the perceived benefits of airflow for thermal comfort and performance during outdoor riding (51–53). The body maps revealed that the neck/upper back, underarm, and chest are the most affected areas by heat and sweat that were suitable for targeted cooling, according to the research findings on local cooling and selective head/neck cooling, which prevent muscle warm-up to some extent (54, 55). Importantly, this functionality goes beyond just enhancing comfort: It allows for connected health applications in which cooling could be initiated in future prototypes when empirically validated physiological or environmental indicators suggest increased heat risk. The integration of such functionalities converts wearable textiles not only into performance enhancement tools but also into health technology devices that are able to detect and react to a threat of heat stress in areas where resources are scarce (6). The incorporation of these features within human-centred co-design processes guarantees functionality despite limited language proficiency, affordability, and maintenance that may be prevalent in developing nations (44).

The comparison with driver thermal-comfort technologies is useful but should be interpreted cautiously. Researchers (19) demonstrated that adaptive climate-control systems can integrate biometric monitoring, environmental sensing, artificial intelligence, and user-centered comfort personalization. For cyclists, however, these ideas must be translated into lightweight, breathable, washable, low-power, and movement-compatible textile systems that account for high metabolic heat production and continuous sweating. This distinction reinforces the importance of future field testing rather than assuming that thermal-comfort technologies from vehicle contexts can be directly transferred to endurance sport.

### 5.2 The prioritised design attributes in smart cooling sportswear for cycling athletes

It can be noted that the order of preferences shows a clear acceptance hierarchy, starting with the use of smart cooling and perforated ventilation panels, then lightweight cooling kits and basic security aspects such as reflective/illumination features. While physiological monitoring (heart rate, hydration, temperature), among other things, is an aspect that cannot be omitted, it is less crucial than active cooling strategies (56). In particular, participants seemed to prefer the latter over mere monitoring and warning capabilities found in many smart garments used in high-resource settings (44).

Acceptance is based on usability issues, which include localized user interface, simple language instructions, and minimal complexity visualization that is relevant for following while working hard, as recommended by sports tracking systems' usability research (35). Aesthetic preferences were related to functional needs (for instance, light colors were preferred due to reflecting heat energy); this was observed in other cultural communities (31).

A possible explanation for preferring cooling over monitoring could lie in lower confidence in sensor accuracy, unfamiliarity with digital wearables, and uncertainty in decoding intricate measurements while working out findings that have been established through usability research in sports (35). On the other hand, perhaps, under hot and humid field conditions, the practicality of cooling could simply carry more weight than gaining access to any further information. Both of these interpretations will be examined in prototype tests by studying preference trends as athletes engage with sensor-triggered cooling, hydration warnings, and user interfaces (56).

In addition, translating wearable heat-health concepts into practice requires attention to implementation pathways beyond garment design. Recent health-impact assessment research highlights that health-protection strategies are more effective when supported by clear triggers, practical guidance, stakeholder engagement, and monitoring arrangements rather than policy recognition alone (57). This perspective is relevant to smart sportswear because evidence from future validated prototypes could inform sport-governance guidelines, public-health messaging, event-level heat-safety protocols, and climate-adaptation strategies for endurance sport in hot climates.

Beyond garment-level design, the findings suggest that wearable heat-health technologies should be considered within broader implementation pathways for athlete safety, sport governance, and climate-adaptation planning. Work on health-impact assessment indicates that effective health-protection strategies require clear implementation triggers, practical guidance, stakeholder engagement, and monitoring processes (57). In endurance cycling, future validated smart sportswear could support event-level heat protocols, club-level procurement decisions, athlete-safety guidelines, public-health messaging, and sport-governance recommendations for training and competition in hot climates. However, this translation requires evidence from prototype testing, physiological validation, usability assessment, implementation feasibility studies, and engagement with cycling clubs, event organizers, sport authorities, health professionals, and local manufacturers.

### 5.3 Limitations and strengths

This exploratory co-design study has several limitations. First, convenience sampling through sports organizations, cycling clubs, and endurance-event networks may have introduced selection bias. Participants were active semi-professional endurance cyclists and may have had higher endurance tolerance, competitive experience, and familiarity with sports technologies than recreational cyclists. Therefore, the findings should be interpreted as design insights from this specific group rather than as representative of all Sri Lankan cyclists. The sample was also highly gender-imbalanced, with 19 male participants and one female participant. As a result, body-zone priorities, garment fit, bib-strap comfort, chest-area ventilation, compression/recovery features, safety visibility, and cultural usability findings should be treated as preliminary and primarily reflective of the study sample. Future studies should recruit across recreational, amateur, elite, regional, and female cyclist populations and examine gender-specific requirements for fit, cooling-zone placement, comfort, modesty, and safety.

Second, the study generated conceptual sketches, design requirements, and qualitative prioritization outcomes rather than a tested digital-health intervention. No functional prototype was developed or evaluated, and no physiological monitoring, thermal-comfort measurement, usability testing under cycling conditions, or heat-strain validation was conducted. Accordingly, the findings reflect participants' perceived needs, priorities, and expectations, not measured reductions in heat strain, dehydration, or heat illness risk. Future research should develop and evaluate functional prototypes under controlled and field conditions using physiological and usability measures such as skin temperature, heart rate, sweat loss, hydration status, perceived thermal comfort, garment fit, interface comprehension, and the reliability of sensor-triggered cooling and alerts.

Third, although individual sketching was used before group discussion to elicit diverse ideas, the group-based nature of the workshops means that some prioritization outcomes may have been influenced by more vocal participants. In addition, the screening process was designed to identify common design directions and participant-perceived value rather than to produce statistical rankings. Future studies could reduce this risk by combining individual ranking, anonymous voting, subgroup analysis, and consensus discussion before final concept selection.

## 6 Conclusion and future work

This study demonstrates how human-centred co-design can inform the development of wearable digital health solutions for heat illness prevention in tropical, low-resource settings. By prioritizing smart cooling and actionable health features over monitoring-only functions, athletes emphasized the need for interventions that may help address heat-related discomfort and perceived heat-risk concerns, subject to future validation. These insights extend smart garment research beyond performance enhancement by positioning smart sportswear as a potential connected health intervention integrating real-time alerts, localized interfaces, and emergency communication. The findings contribute to the mHealth and human factors literature by highlighting usability, cultural adaptation, language localization, simplified data visualization, and cost-sensitive design as key conditions for technology acceptance in developing countries. Future work should focus on prototype development, physiological and usability validation under cycling conditions, component-level feasibility assessment, and stakeholder engagement to determine whether these concepts can be translated into implementable heat-protection strategies. By embedding digital health functions into culturally responsive sportswear, this research advances equitable access to connected health technologies for underserved athlete populations.

## Data Availability

The raw data supporting the conclusions of this article will be made available by the authors, without undue reservation.
