# Supplementary material for: Value driven co-design of smart sportswear for heat illness prevention in tropical regions
Source: Front Digit Health. 2026 Aug 18;8:1872925. doi: 10.3389/fdgth.2026.1872925 (PMC13529768; doi:10.3389/fdgth.2026.1872925)
Supplement: Supplementary file 1 [file Datasheet1.pdf]

Table A : Co-design sketches

ID	Garment	Cooling concepts	Sketch
1	T shirt	Wind cooling (Evaporative /perforated material)	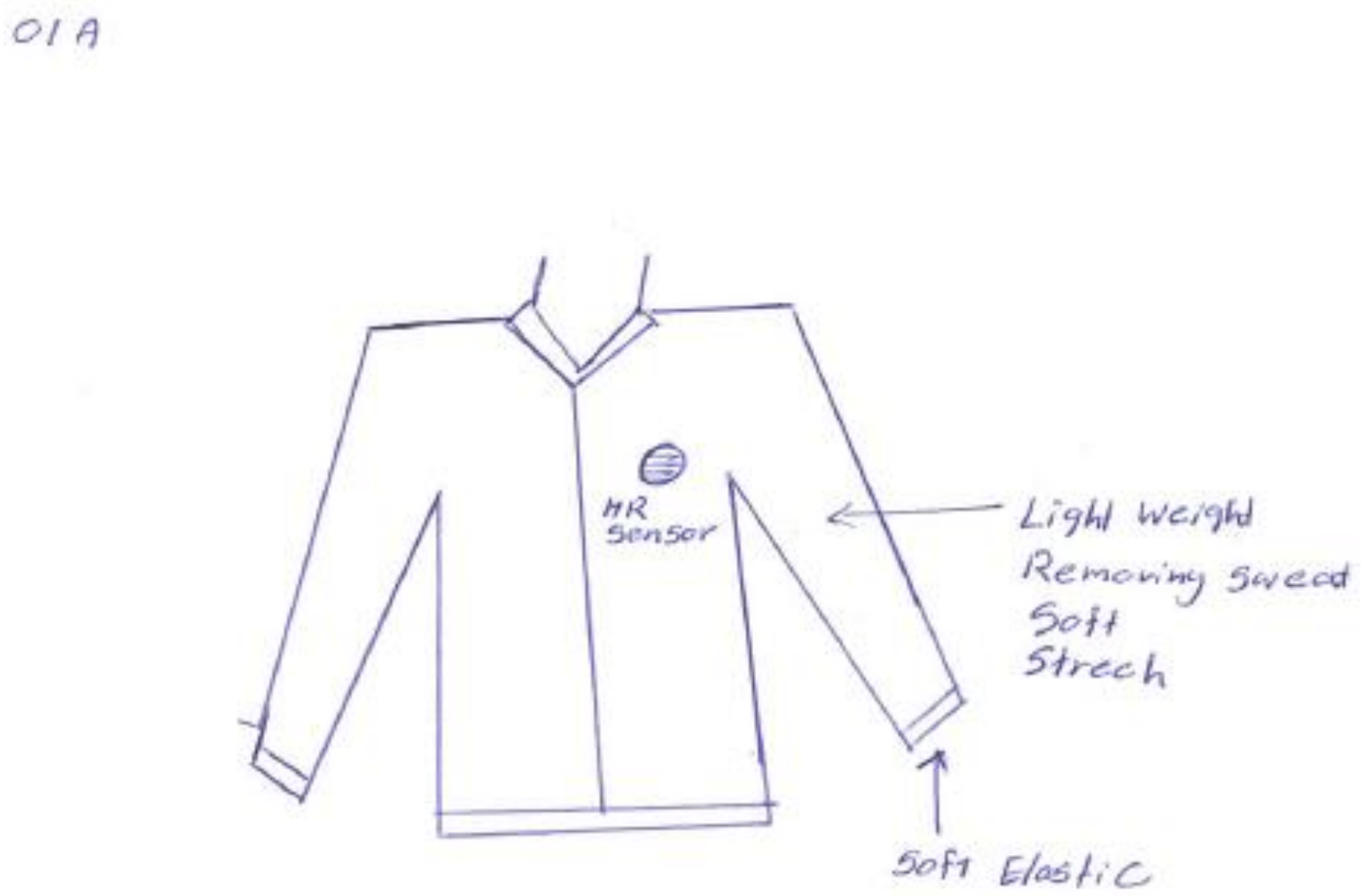

2	T shirt	Post cooling-cooling material	02 A<br>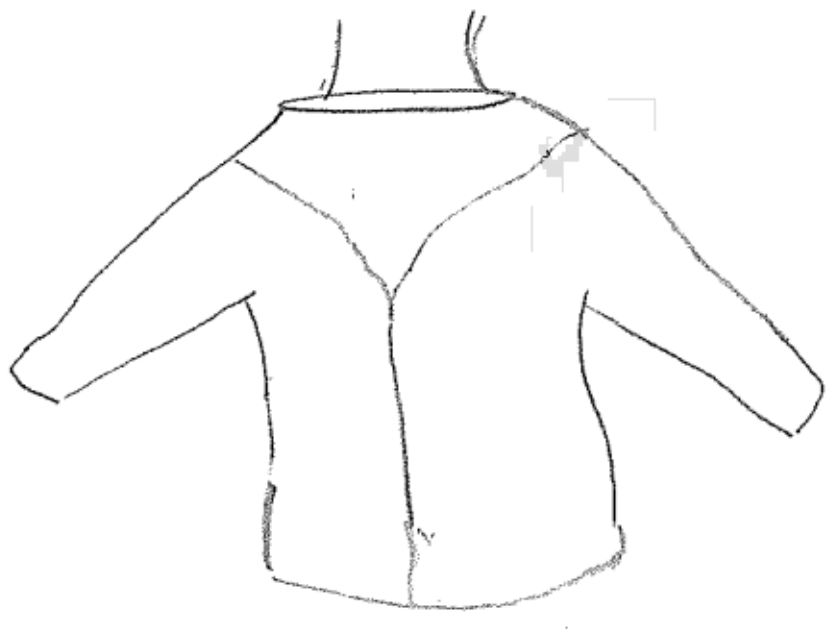<br>*. තරඟයෙන් පසුව ඇඟ සිසිල් කිරීම .<br>* සැහැල්ලු රෙද්දක්

3	T shirt	No	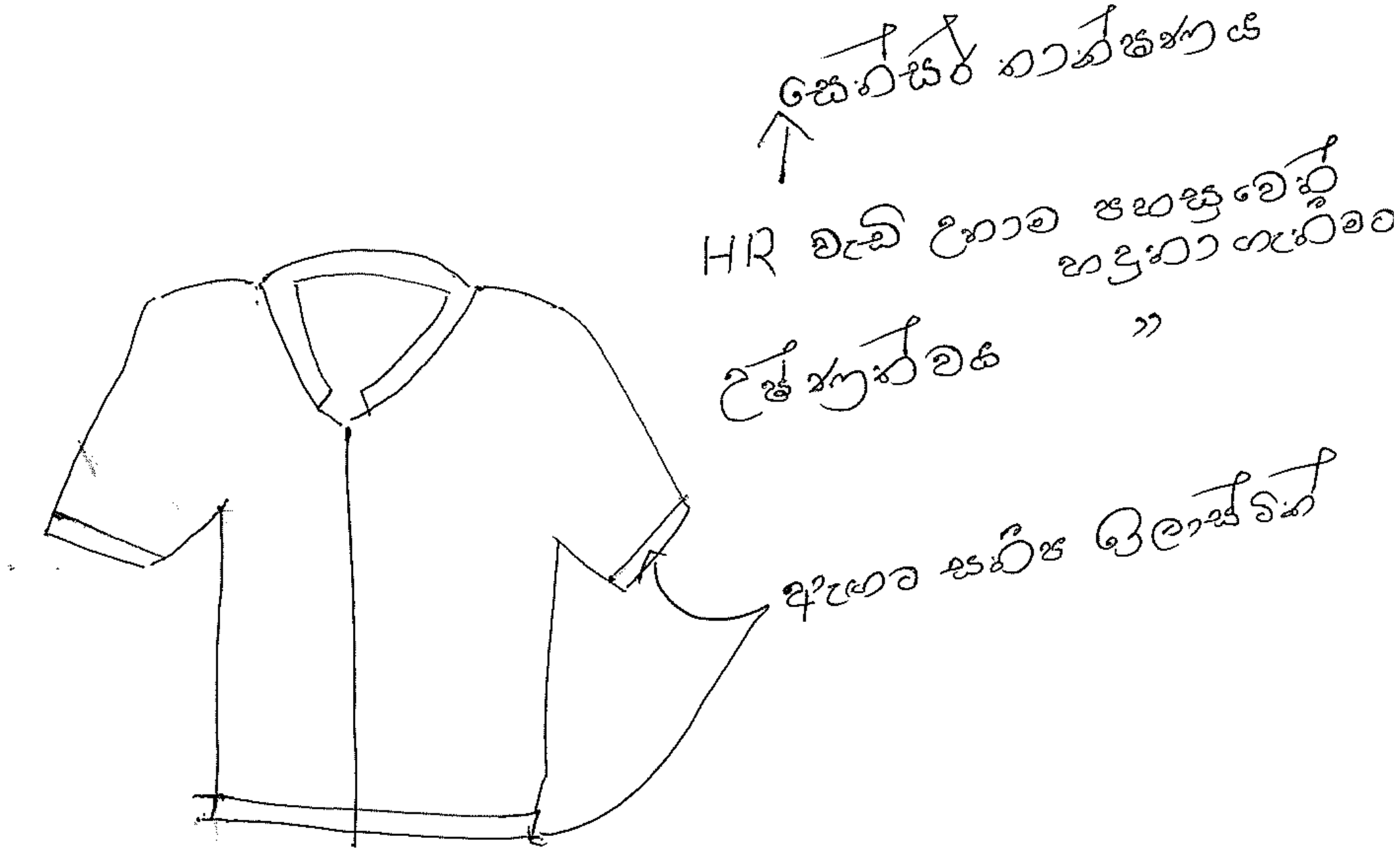
4	T shirt	Wind cooling (Perforated material insertions, Open construction)	

5	Shorts	Wind cooling (Perforated material insertions)<br>Cool gel pack	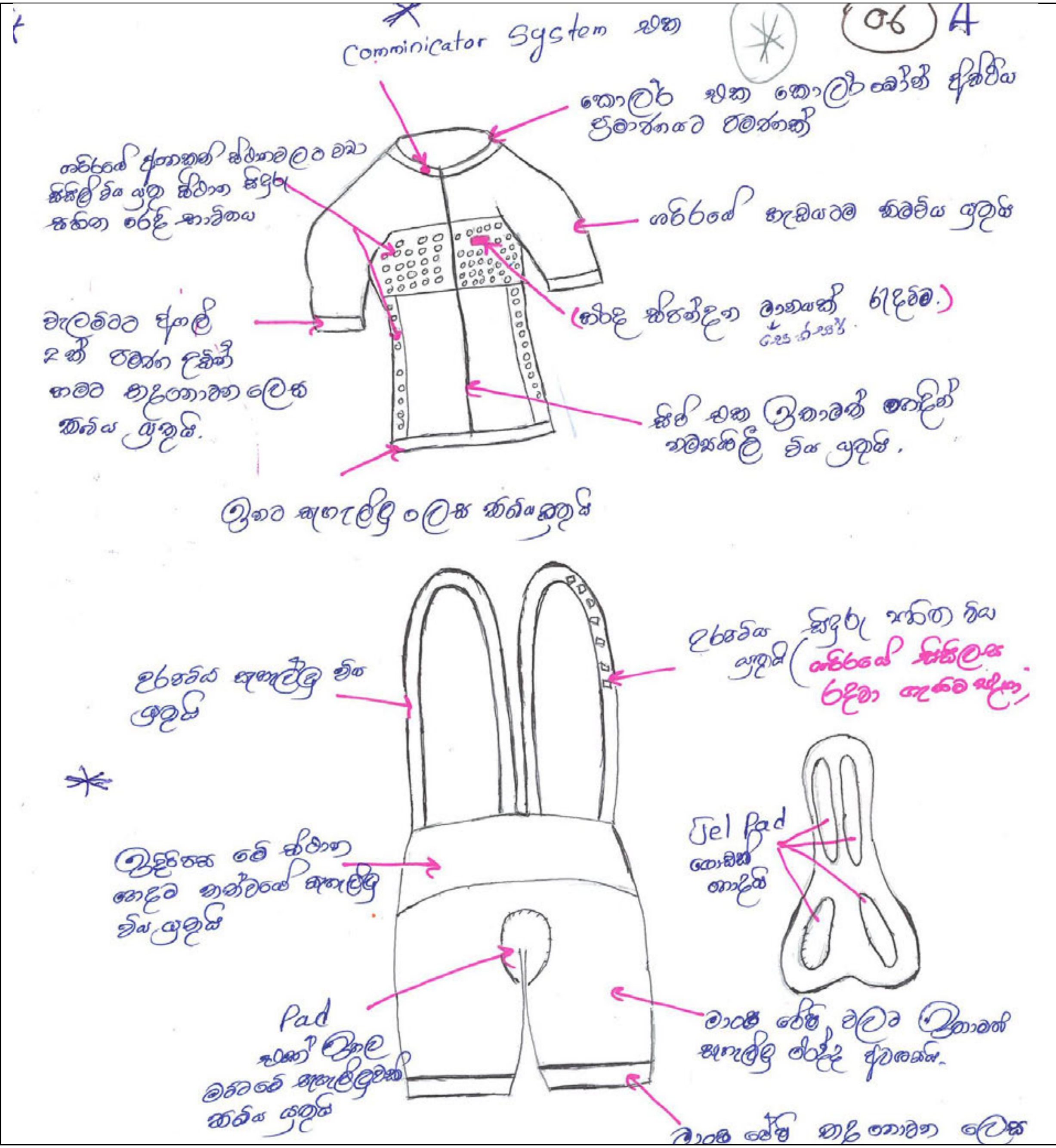

6	T shirt	Wind cooling (Perforated material insertions)	(07A)<br>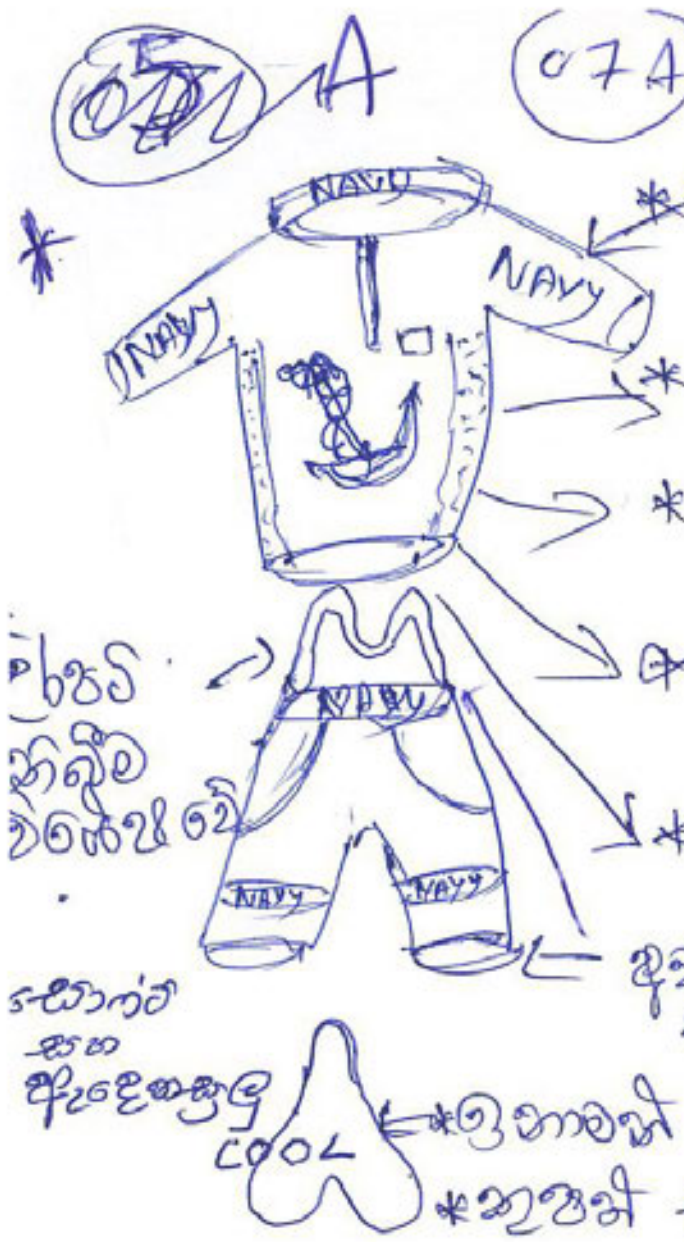<br>* මුලු Jercy එකම ඇදෙන සුළු ඇගට පහසු රෙද්දකින් නිමවියයුතුයි.<br>ඉස්ර එක Haft එකක් වීම.<br>* ඇගේ ප්‍රමාණයටම තිබීම ඉතා වැදගත්.<br>* කොලර් තිබීම හෝ නැතිවීම ක්‍රීඩකයාගේ කැමැත්ත පරිදි සිදු වේ.<br>* Hard moniter belt එක රඳවාගැනීමට පහසු ~~Pocket එකක්~~ විදිහට තිබීම<br>* මයික් එක රඳවා ගැනීමට වෙනම Pockt එකක් තිබීම.<br>අවසාන කොළර් ඉලාස්ටික් පටිය වෙනුවට පෝරේ රෙද්දෙන්ම නාටිය නිමවීම ඇතුලට මදක් කදවන පරිදි. ~~[illegible]~~<br>* ඉතාමත් පහසු සිනිදු මතුපිටකින් යුක්ත වීම.<br>* ඉඳුන් සහිත ජෙල් මතුපිට සහිත. (~~[illegible]~~<br>* ක්‍රීඩකයාගේ ශරීරයේ ප්‍රමාණය අනුව සයිසස් වර්ග කල යුතුය<br>උදා (S, m, L)
7	Shorts	Cool pack	
8	T-shirt	Wind cooling (Perforated material insertions)	

9	Shorts	cool gel pack	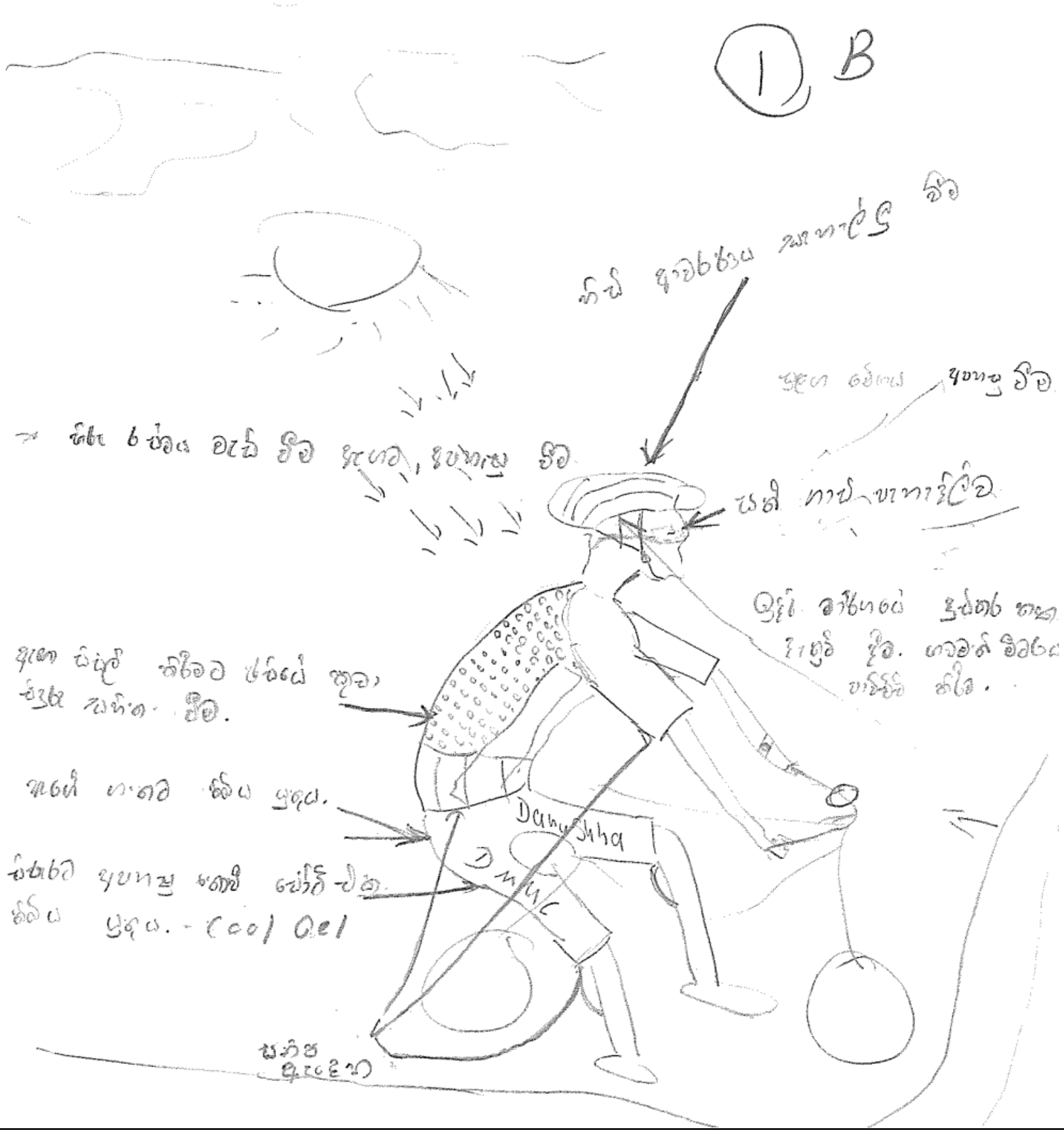
10	T-shirt	Wind cooling (Perforated material insertions) Cool get pack Cooling material	

11	Shorts	Cooling material<br>Cool gel pack	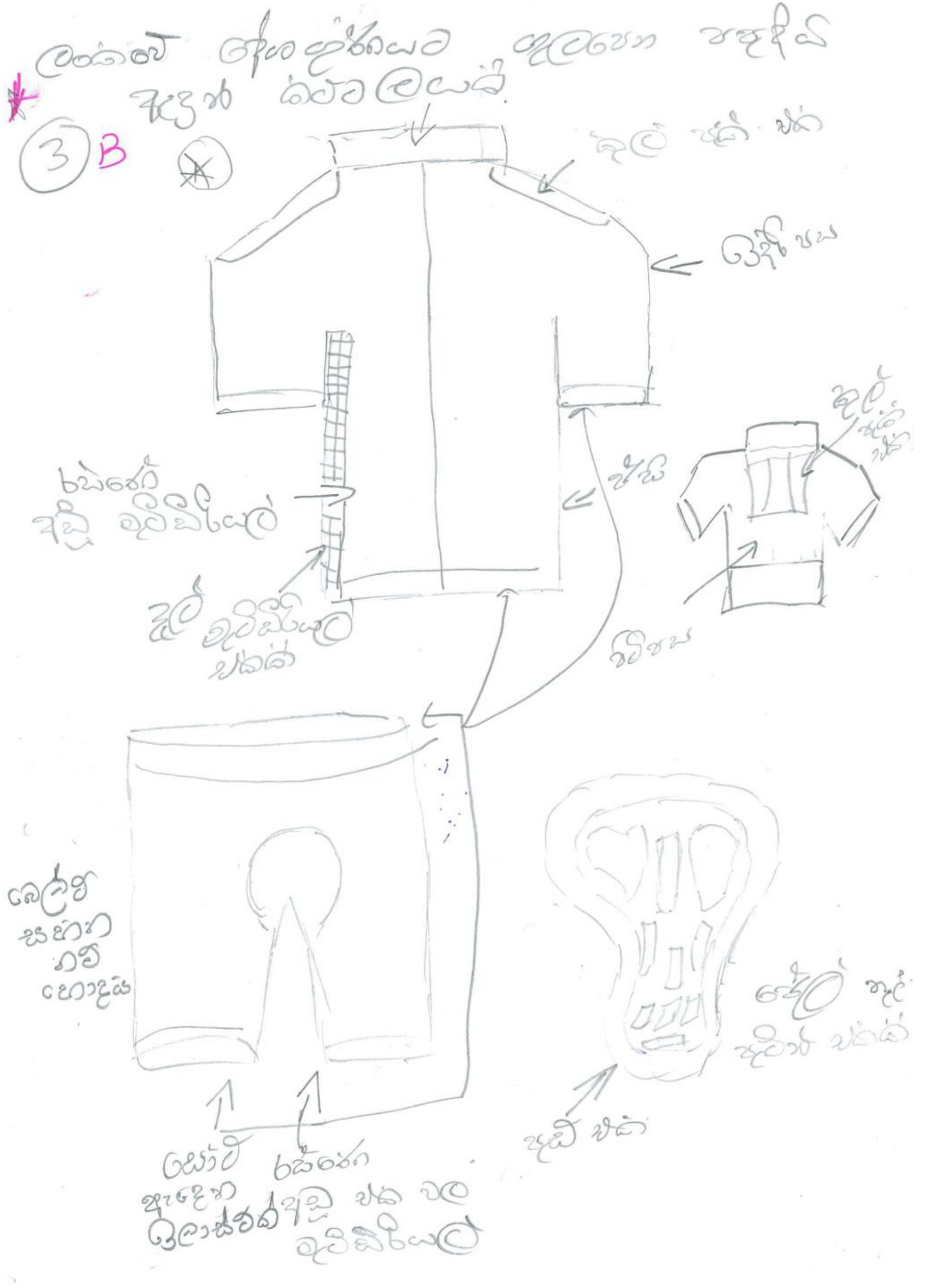

12	T-Shirt	Wind cooling (Perforated material insertions)<br>Cooling material<br>Cool get pack	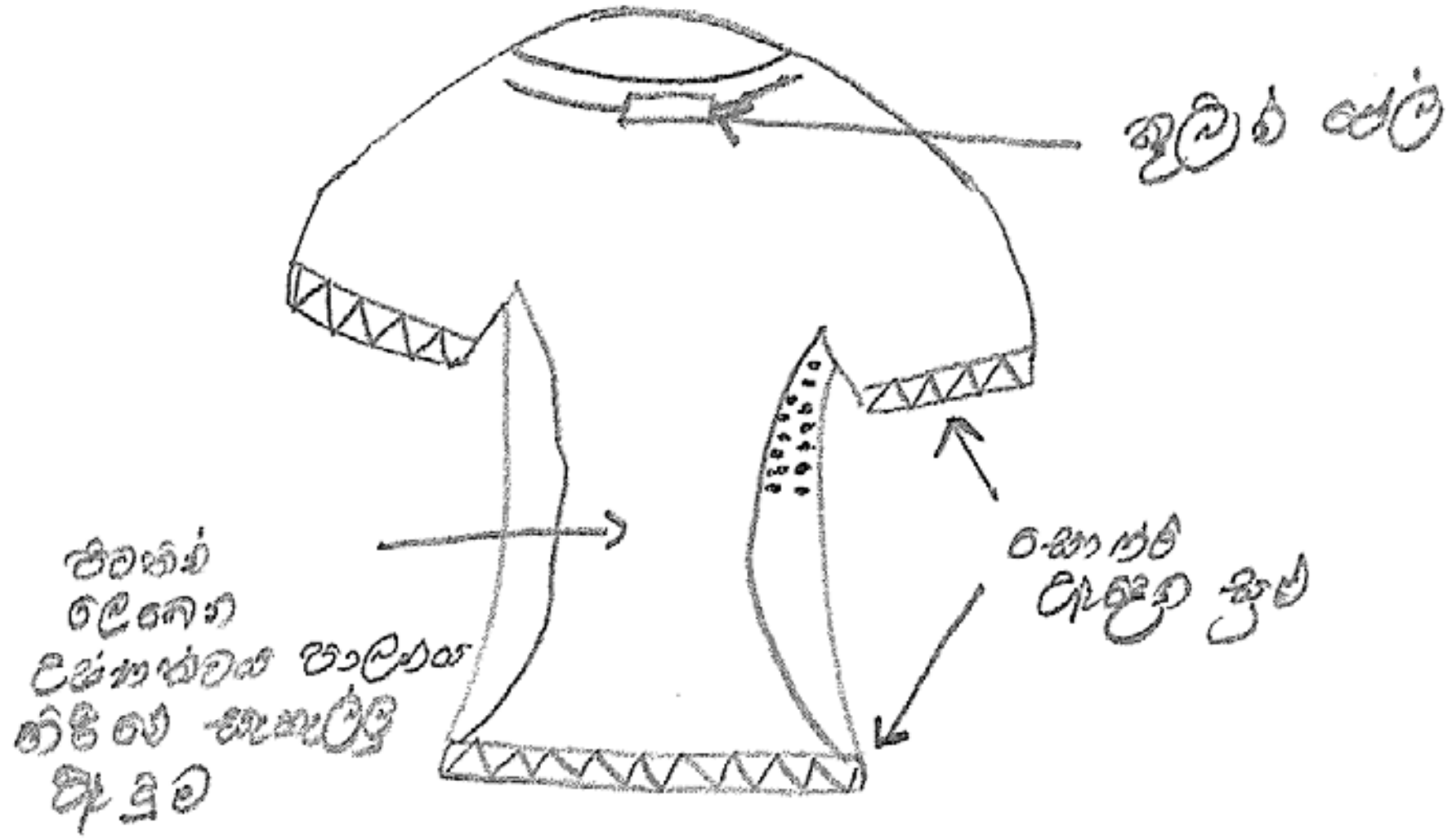

13, 14	Sleeve and Pant	Wind cooling (Holes to remove sweat) Cooling material Sweat absorbing material	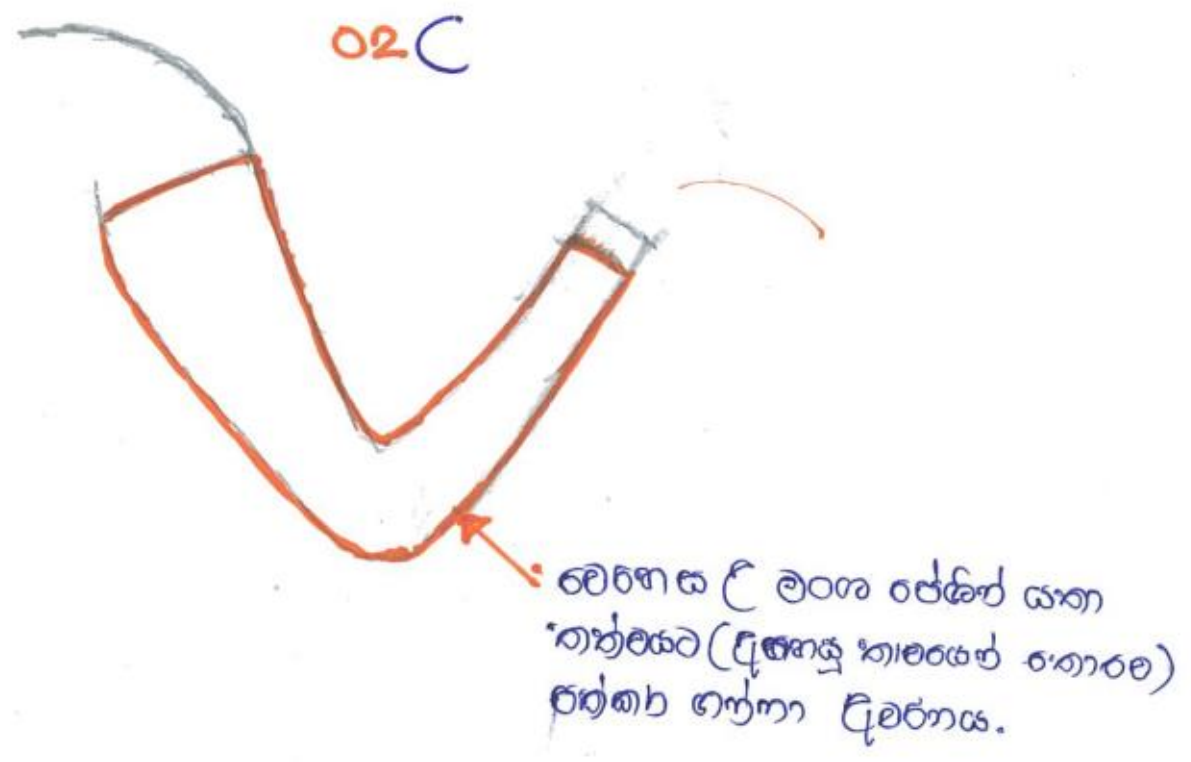 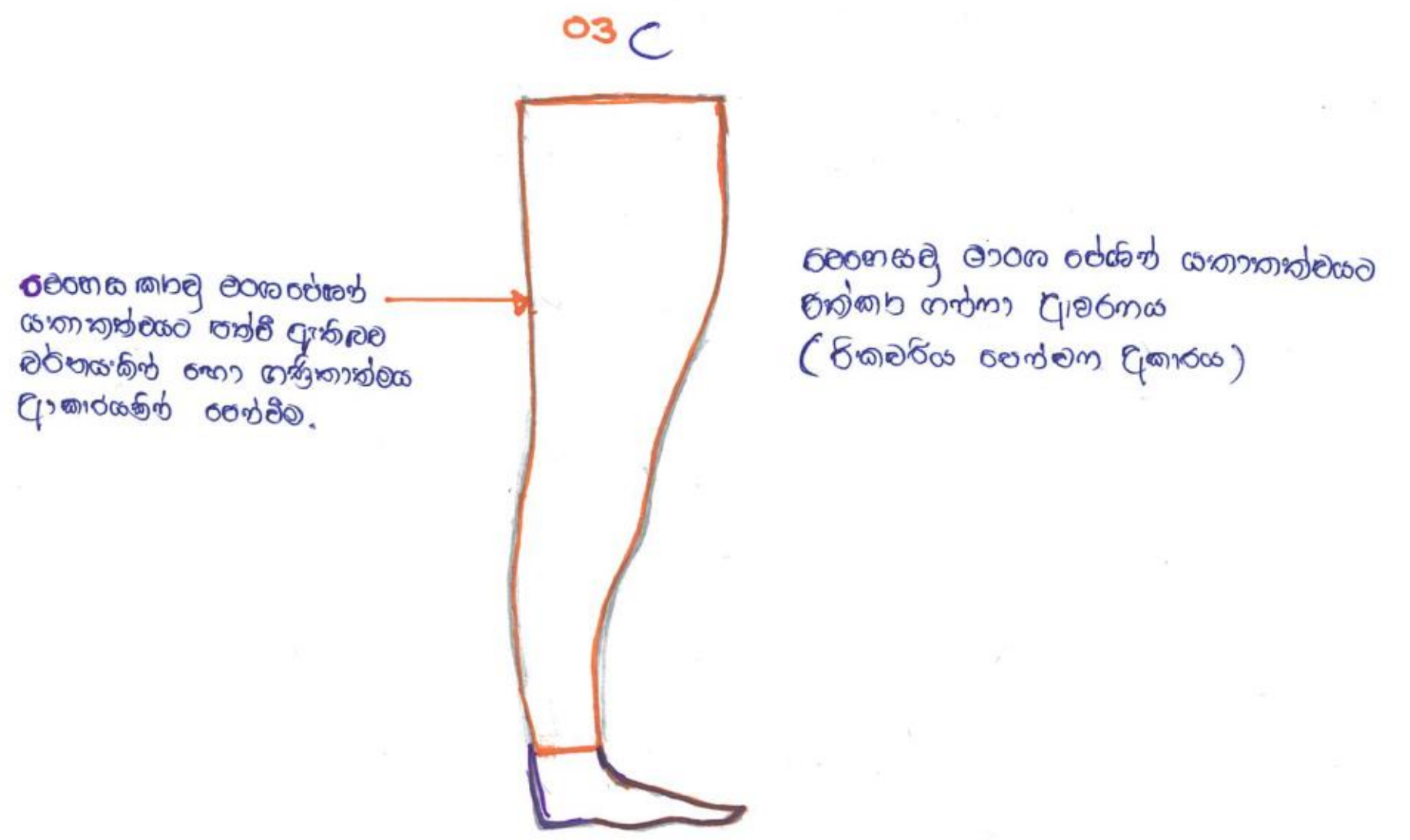

15	Short	Cool get pack	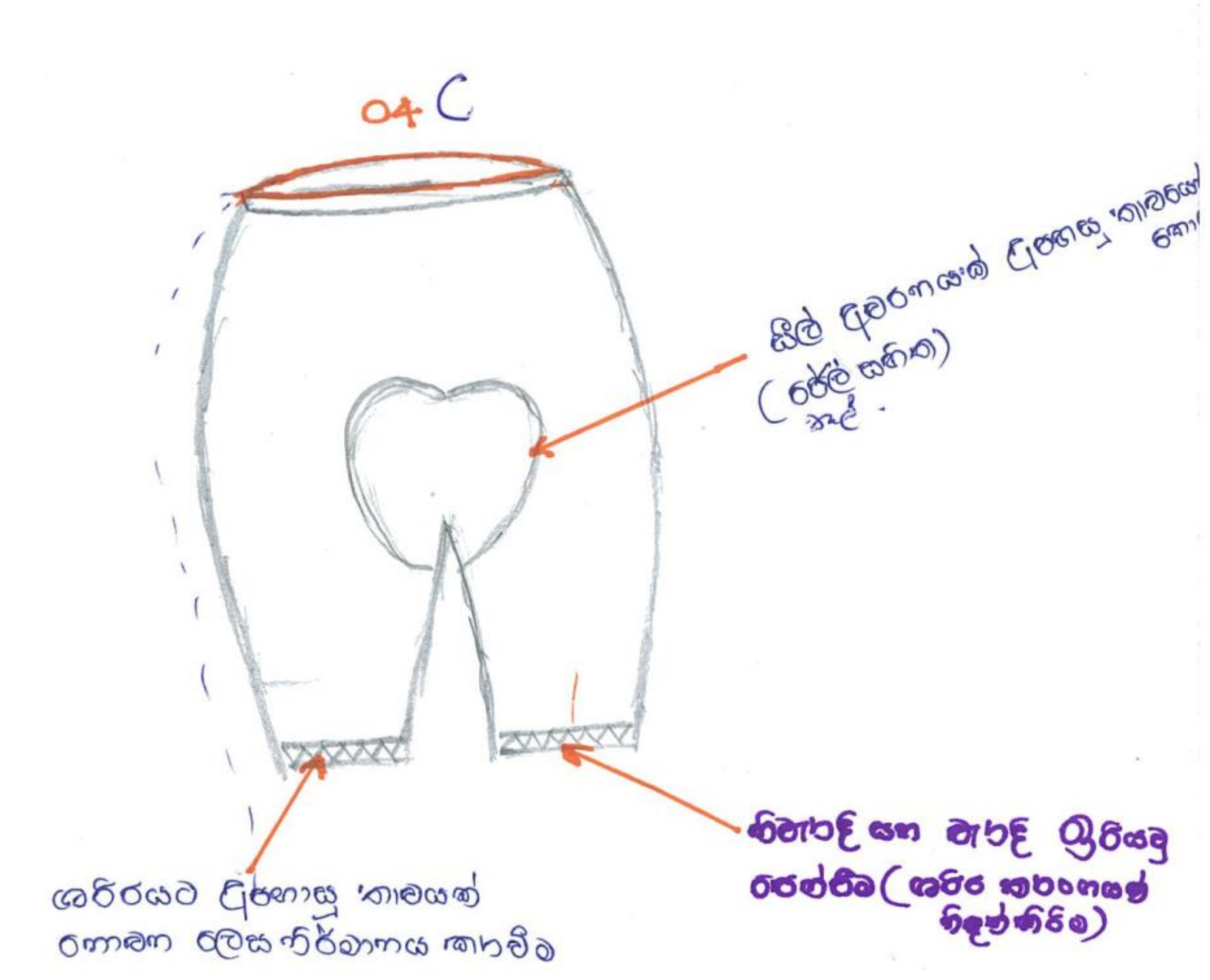

16	T-shirt	Wind cooling (Perforated material insertions) Cool get pack	5C 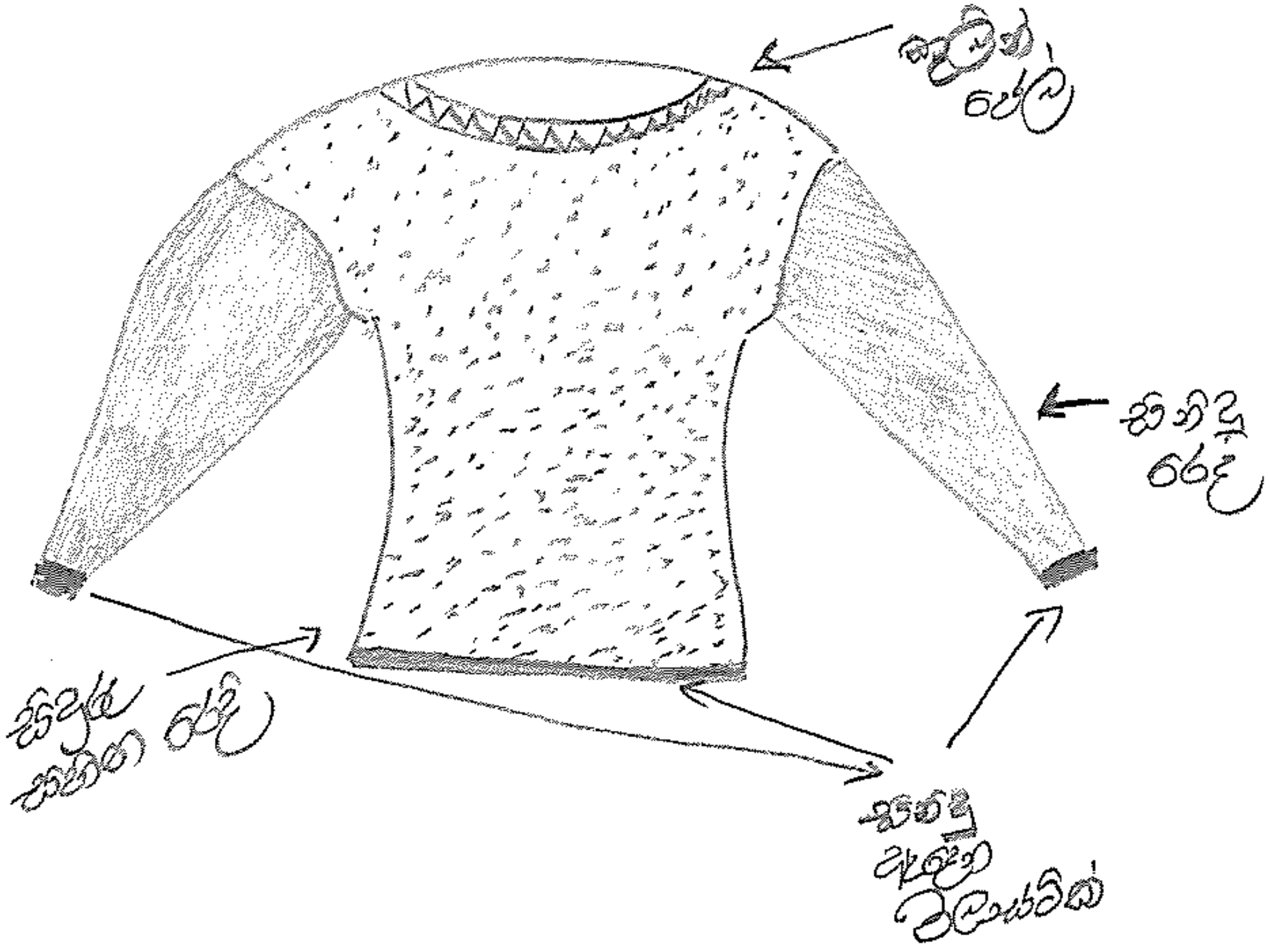
17	T-shirt	Wind cooling (Perforated material insertions)	

18	Shorts	Wind cooling (Perforated material insertions) Cool gel pack	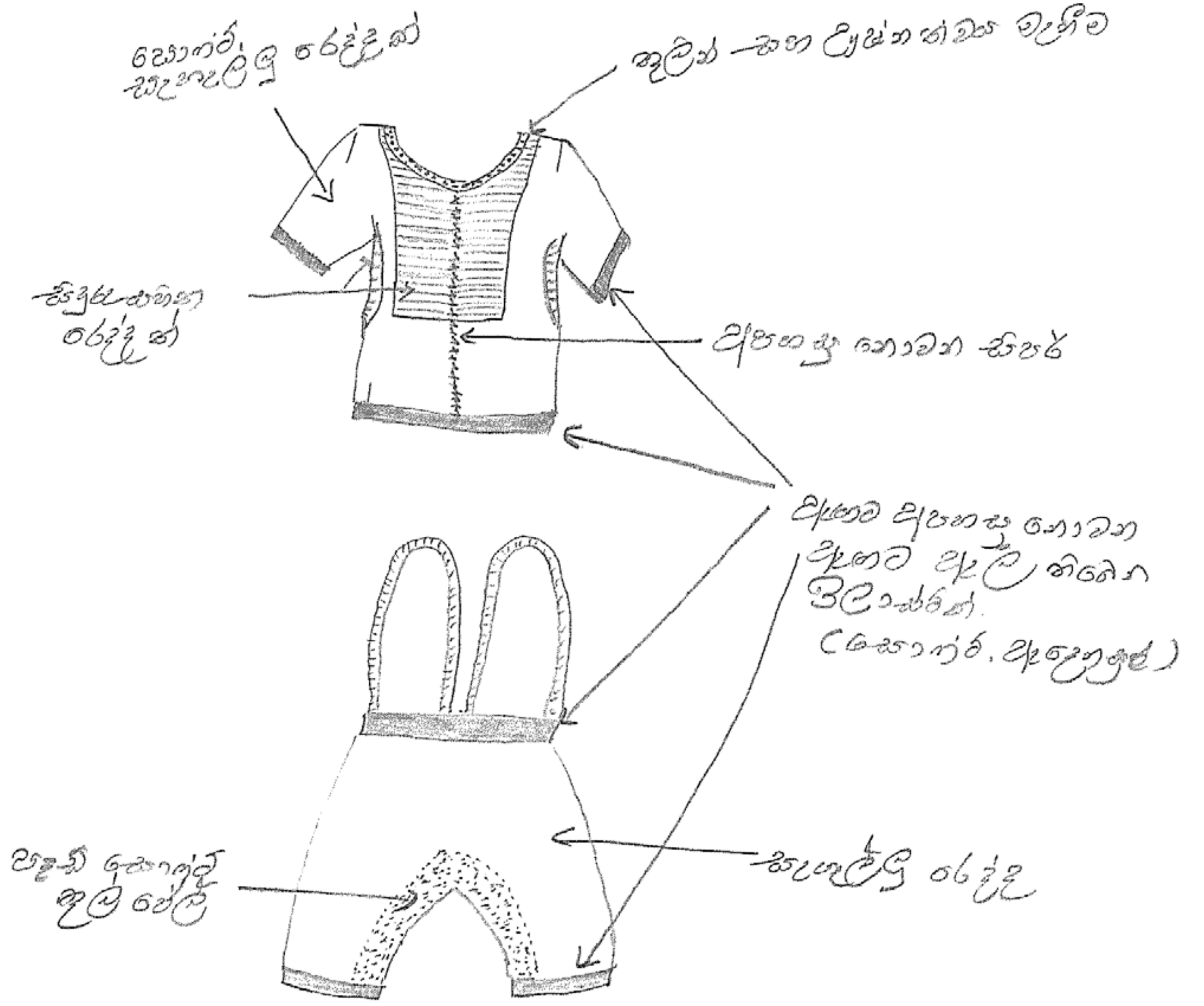

19	Shorts	Wind cooling (Perforated material insertions) Cooling pad	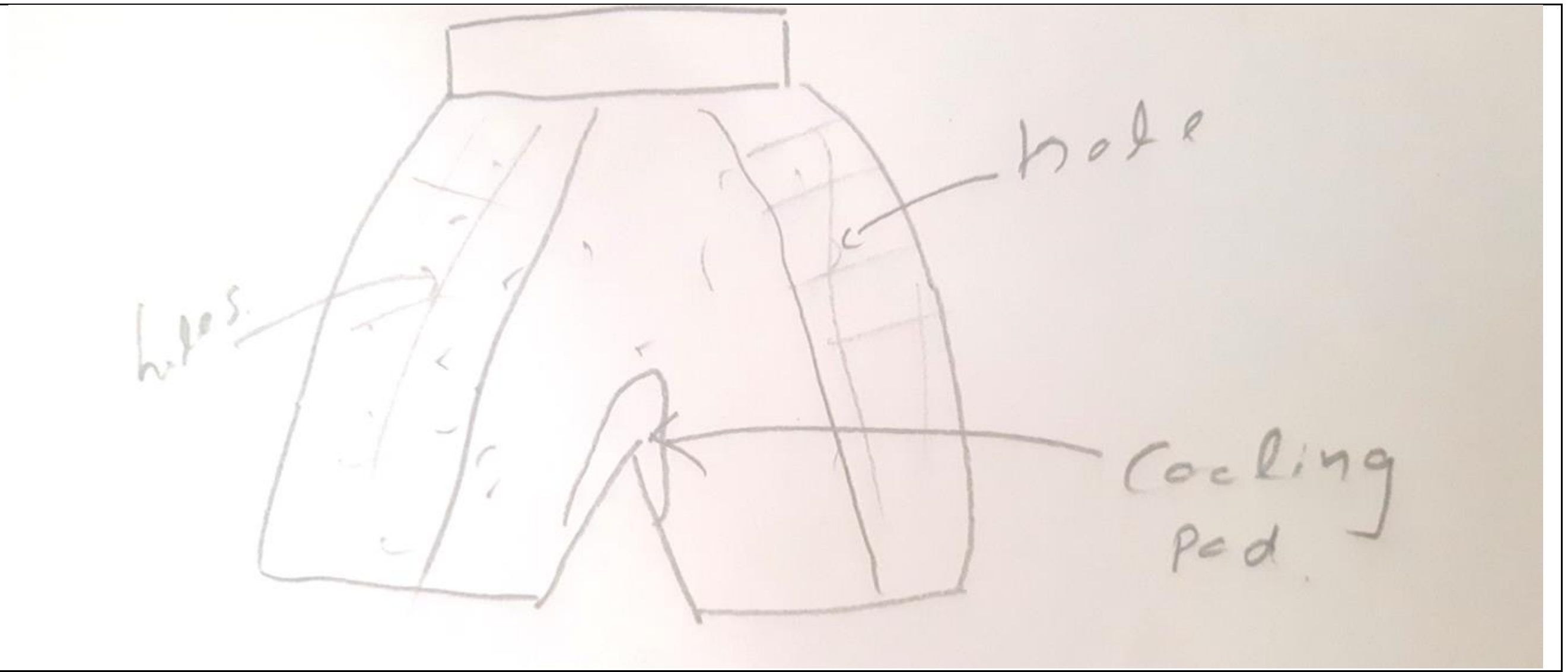

20	T-shirt	Wind cooling (Perforated material insertions) Cooling pack	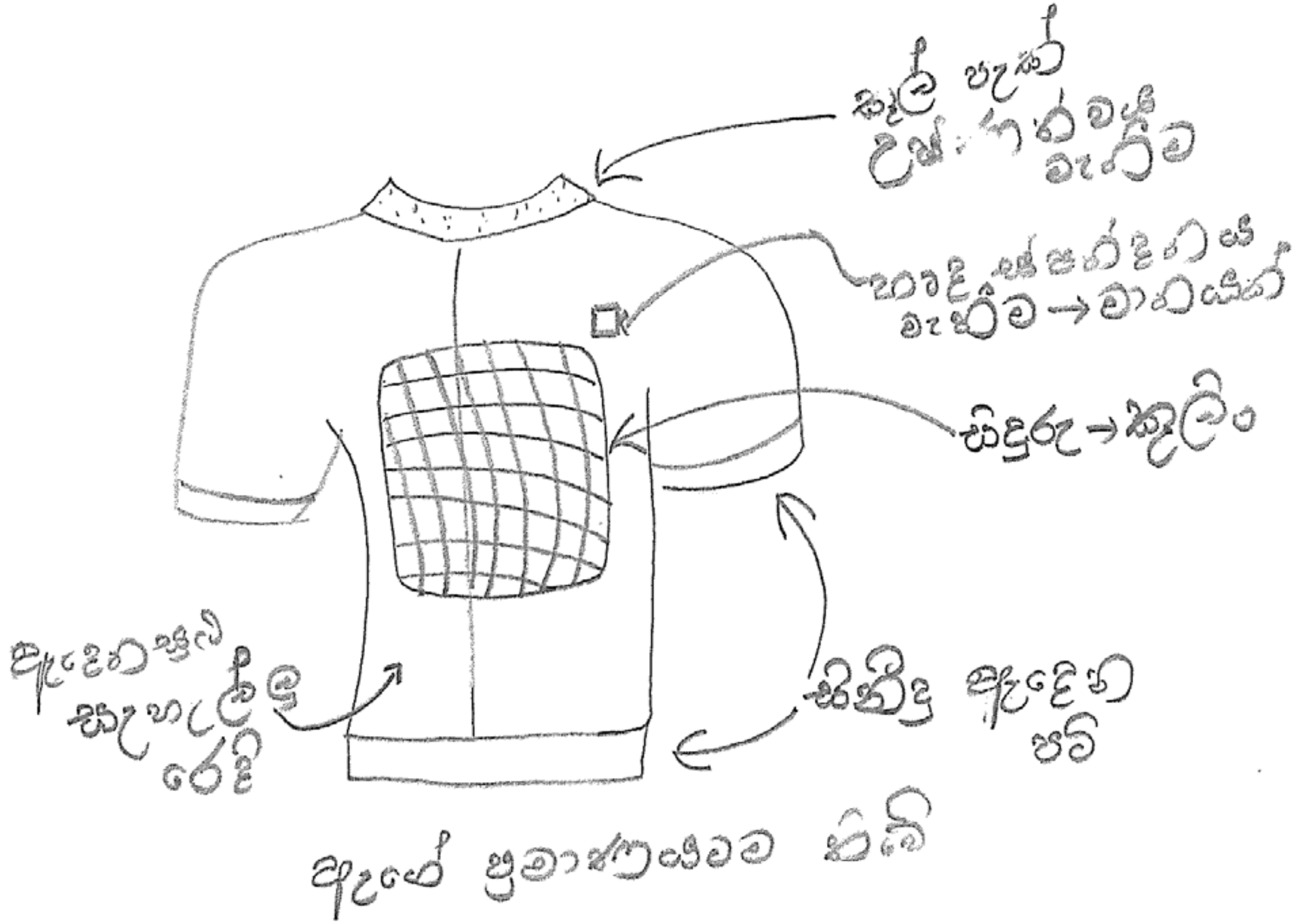

21	T-shirt	Wind cooling (Evaporative /perforated material) Cooling pack	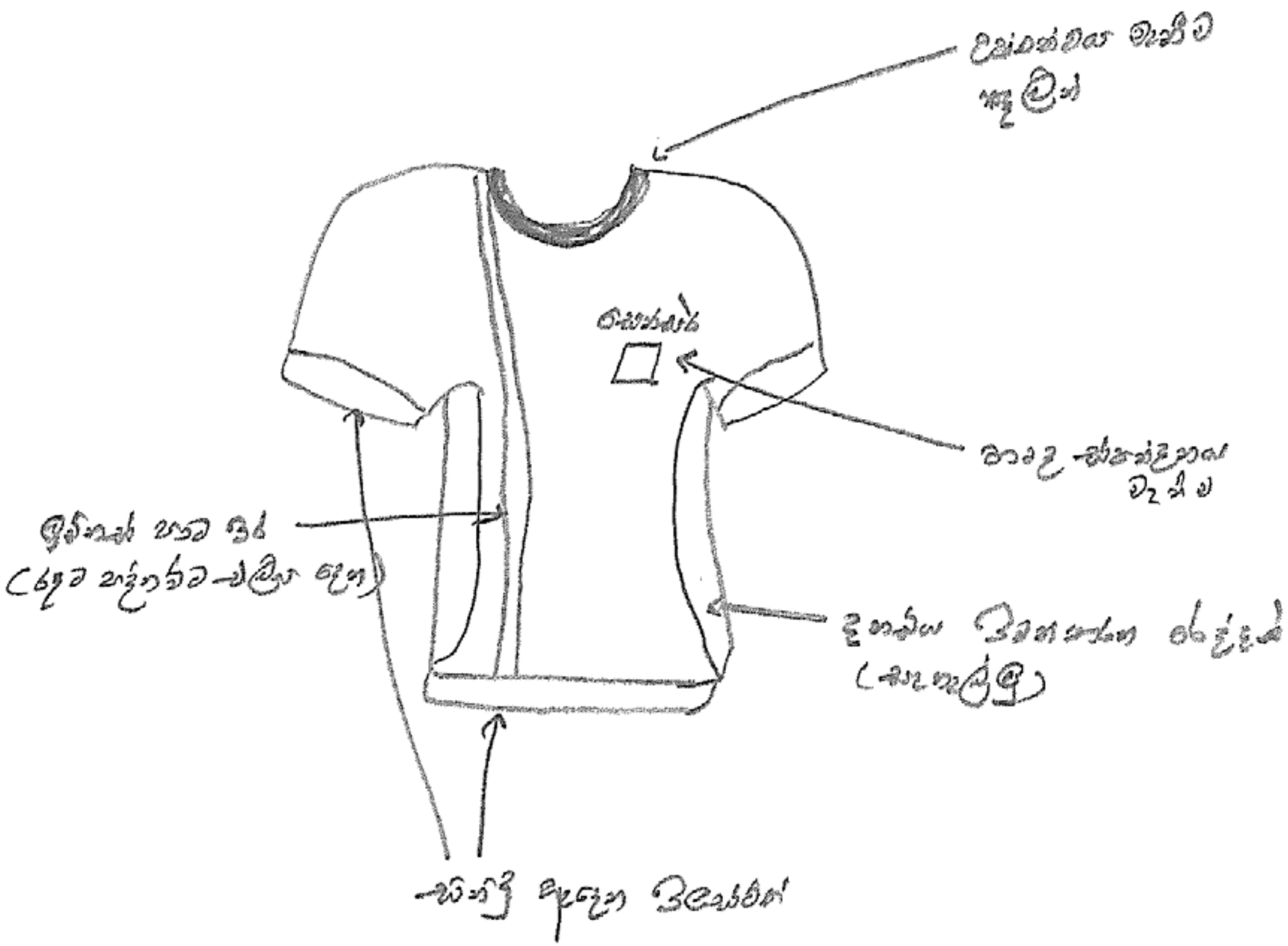
22	Shorts	Wind cooling (Perforated material insertions) Cool gel pack	

23	T-shirt	Wind cooling (Perforated material insertions)	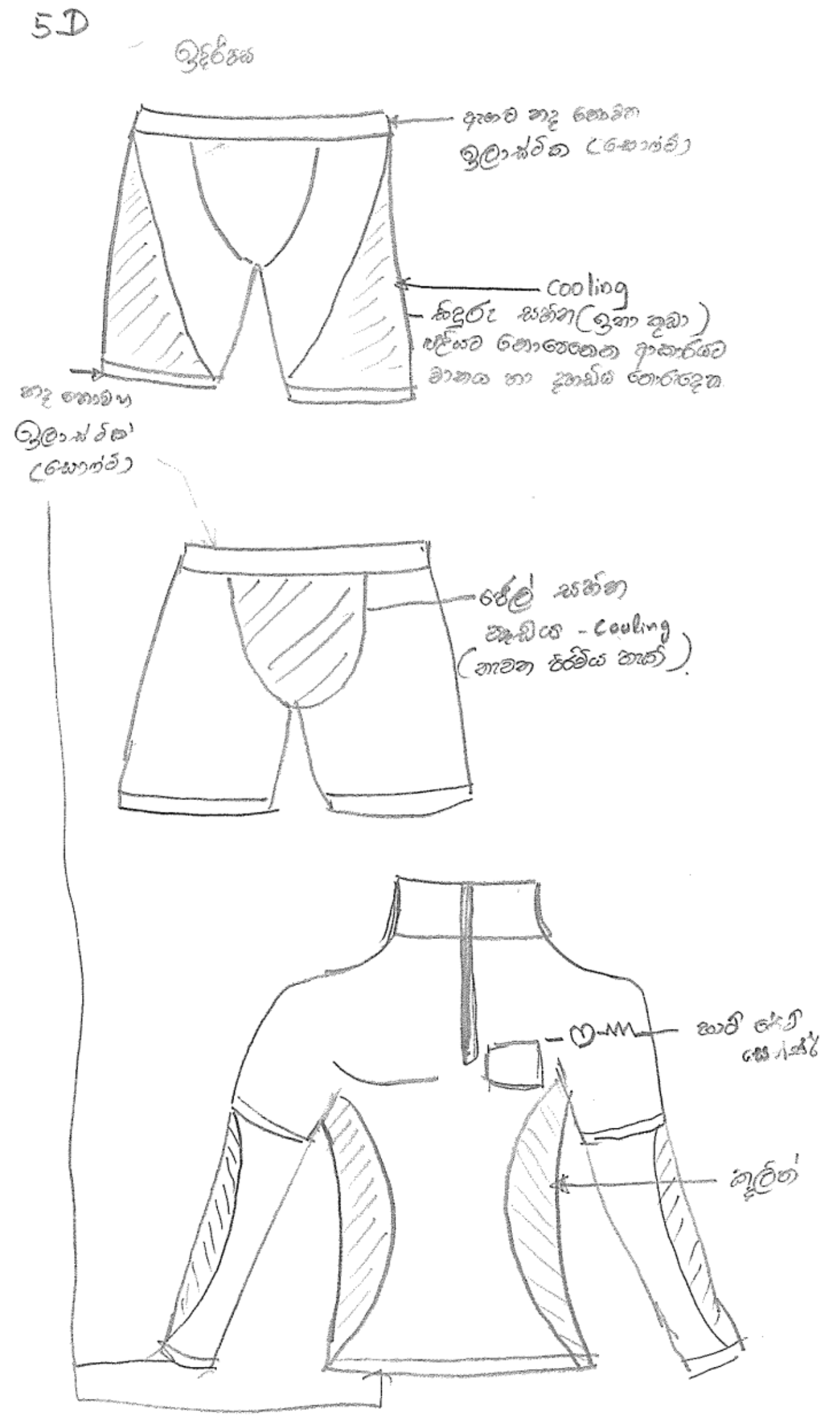

24	Shorts	Cool gel pack	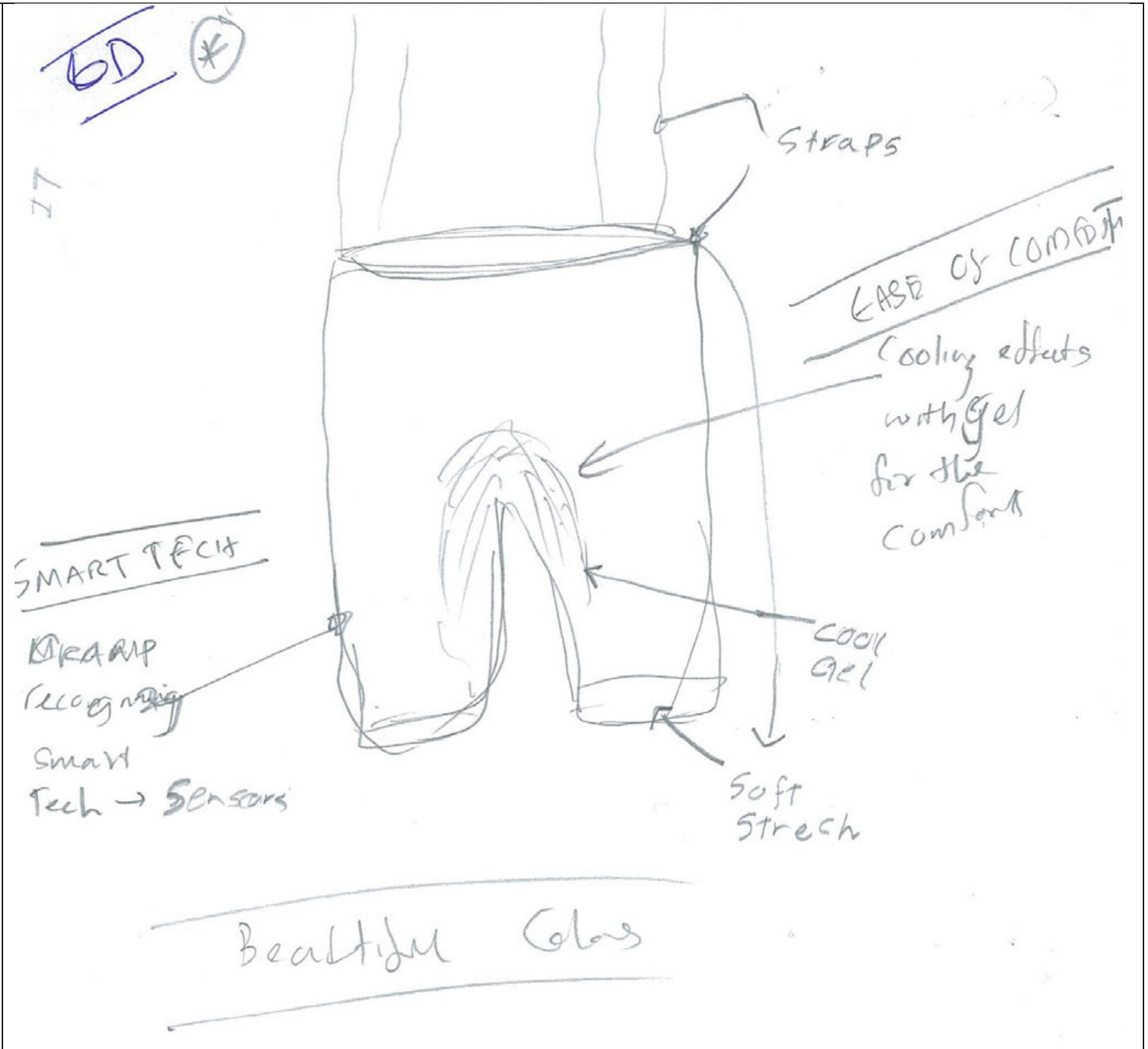

25	T-shirt	Water cooling concept<br>Wind cooling	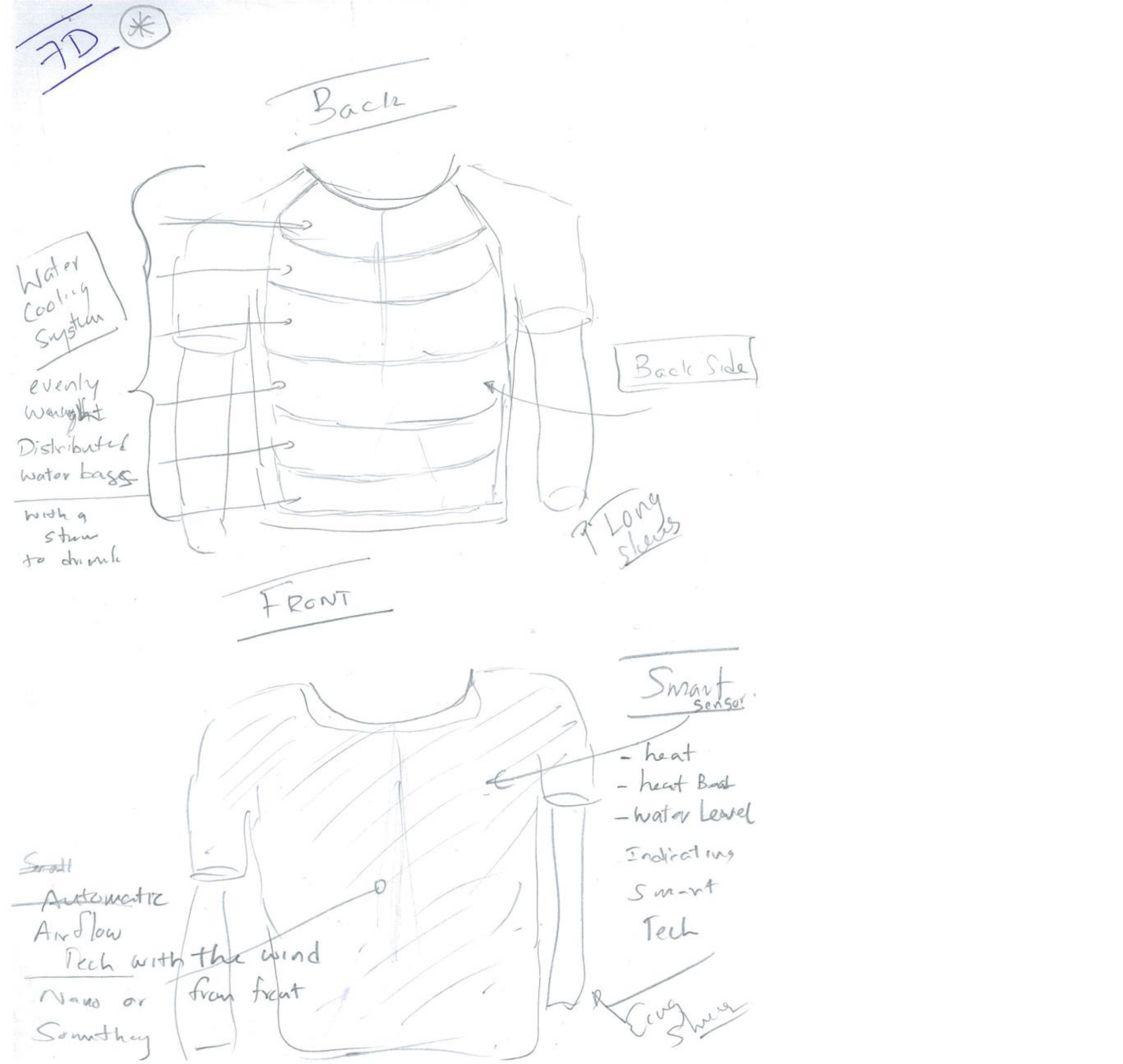

26	T-shirt	Wind cooling (Perforated material insertions) Cool gel pack	

27	Shorts	Cool gel pack	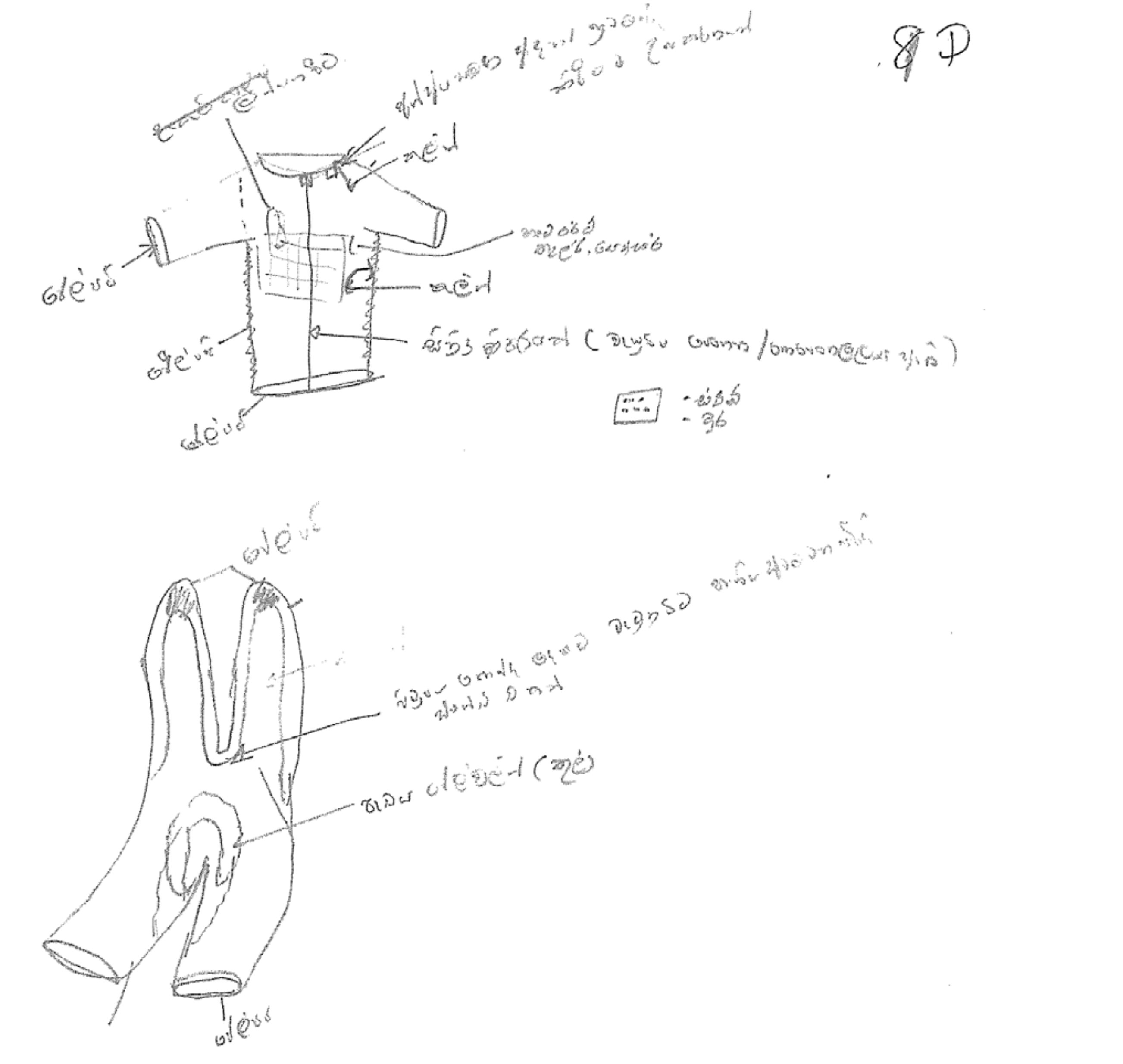

28	T-shirt	Wind cooling (Perforated material insertions)	
29	Shorts	Cool gel pack	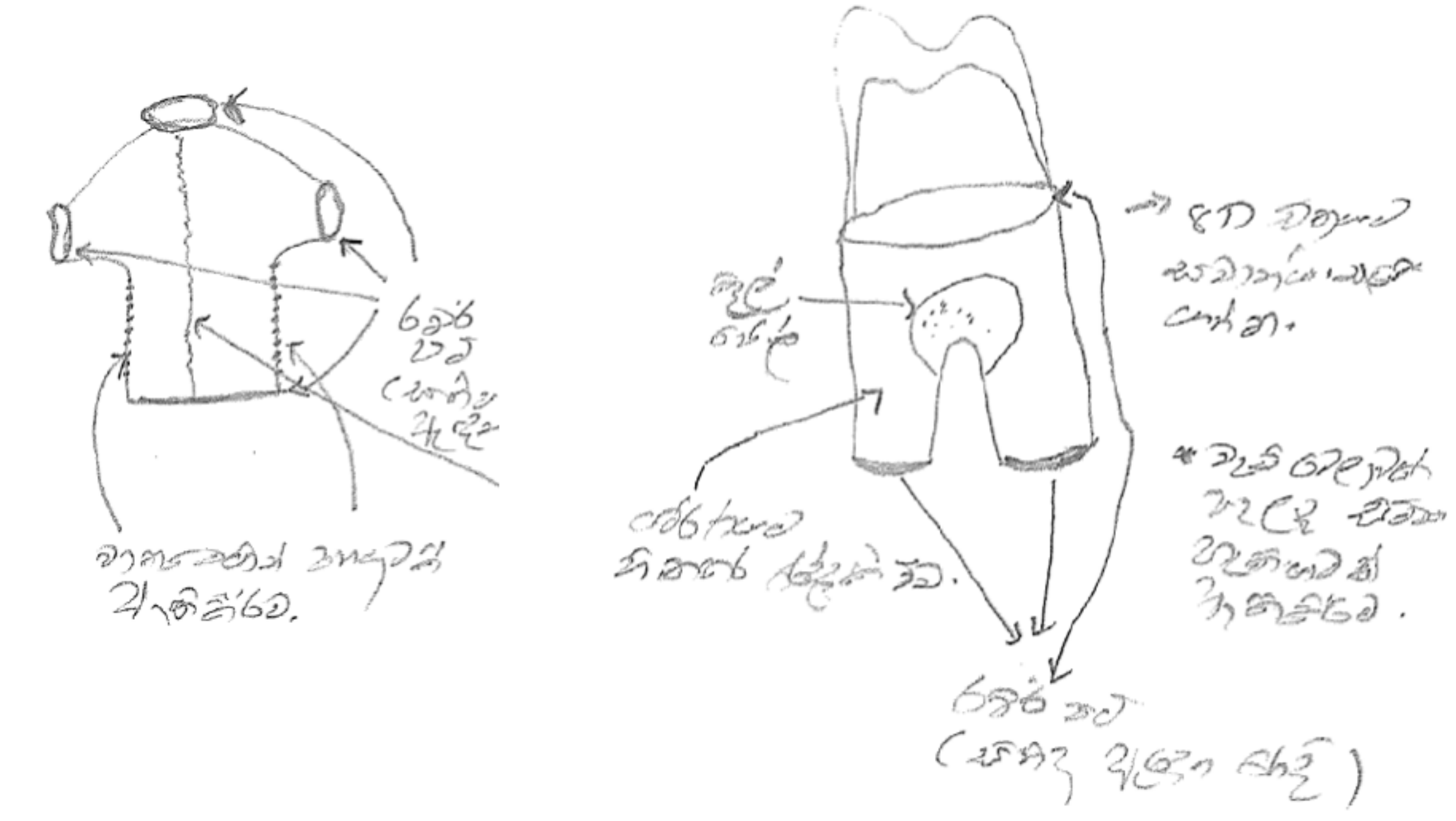
30	T-shirt	Wind cooling (Perforated material insertions)	

31	Shorts	Wind cooling (Perforated material insertions) Cool gel pack	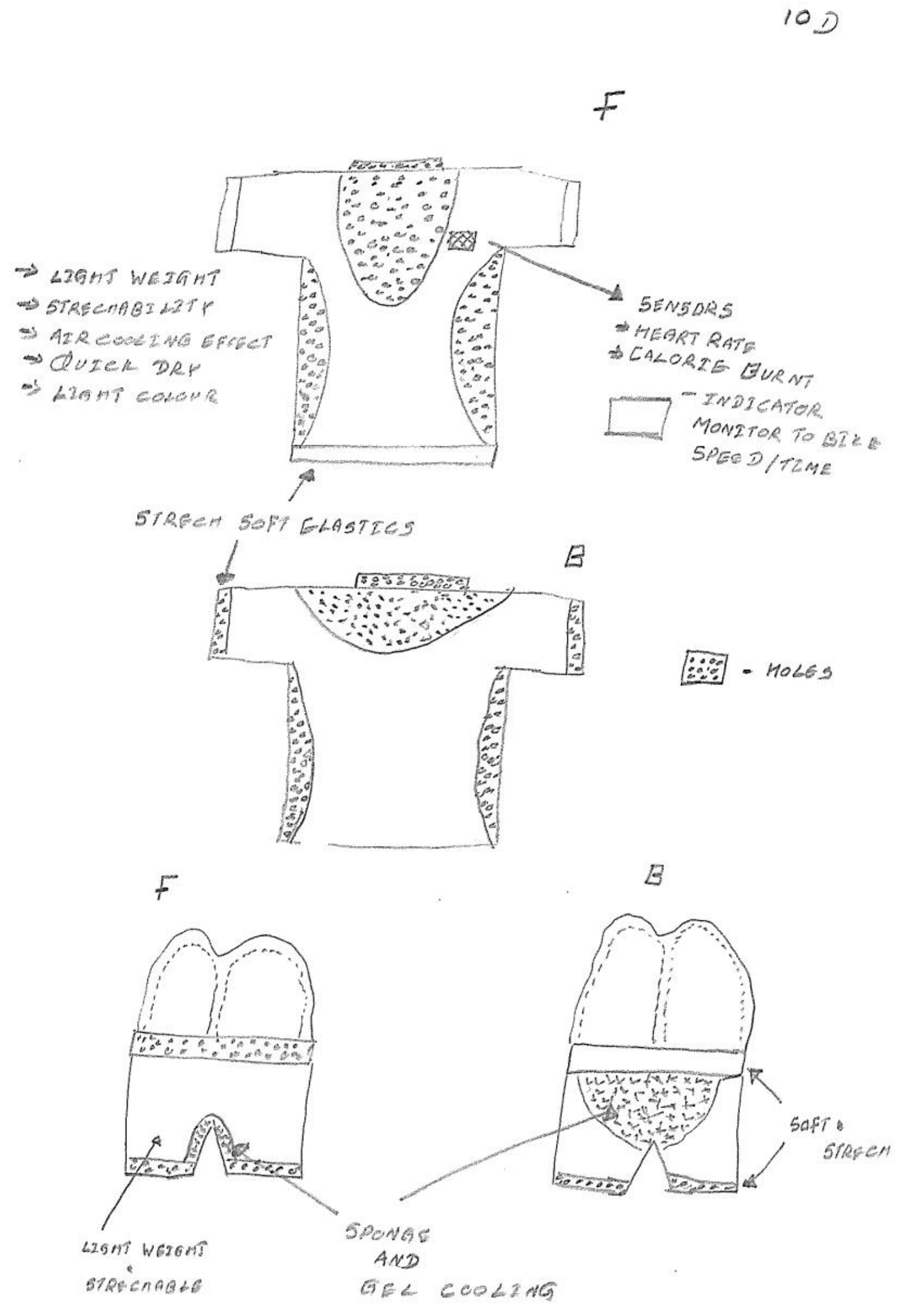
